# Effects of Amphiphilic Chitosan on Stereocomplexation and Properties of Poly(lactic acid) Nano-biocomposite

**DOI:** 10.1038/s41598-018-22281-1

**Published:** 2018-03-12

**Authors:** Arvind Gupta, Akhilesh Kumar Pal, Eamor M. Woo, Vimal Katiyar

**Affiliations:** 1 Department of Chemical Engineering, Indian Institute of Technology Guwahati, 781039 Assam, India; 2 Department of Chemical Engineering, National Cheng Kung University, Tainan, 701-01 Taiwan

## Abstract

This article demonstrates an elegant approach for the fabrication of high heat-stable PLA using an industrially viable technique i.e. melt extrusion, which is quite challenging due to the higher viscosity of poly(lactic acid) melt. scPLA has been fabricated by melt extrusion of PLLA/PDLA using nano-amphiphilic chitosan (modified chitosan, MCH) which has been synthesized by grafting chitosan with oligomeric PLA via insitu polycondensation of L-lactic acid that possibly increases the molecular surface area and transforms it into nano-amphiphilic morphology and in turn lead to the formation of stereocomplex crystallites. The effect of MCH loading on the structural, morphological, mechanical and thermal properties of PLLA/PDLA have been investigated. The modification of chitosan and formation of stereocomplexation in PLA has been confirmed by FTIR and XRD techniques, respectively. Heat treatment has also laid a significant effect on the stereocomplexation and the degree of crystallinity of stereocomplex crystallites has been increased to ~70% for 1.5 wt % MCH content with the absence of homocrystals. The heat deflection temperature is found to be more than 140 °C for the biocomposite with 1.5% MCH in comparison to ~70 °C for pristine scPLA. The biocomposites display significant improvement in UTS and Young’s modulus.

## Introduction

In recent decades, the disposal of petroleum based polymers and plastic waste has become a threat to our environment^1^. Development of biodegradable polymers such as poly(lactic acid) (PLA), poly(β-hydroxybutyrate) (PHB) etc. is one of strategy to overcome this problem. PLA is a biodegradable, bio-absorbable and biocompatible polymer produced from lactic acid or lactide monomers which can be produced by renewable resources such as wheat^2^, corn^3^ etc. Due to its good mechanical strength and easy processability over other biodegradable polymers^4^, it is used in packaging^5,6^, biomedical^7^ and some engineering applications. The stereocomplex polylactic acid (scPLA)^8,9^ which is a special crystalline arrangement between enantiomeric PLAs^10^, has been an attraction for the researchers and studied extensively^11^. Due to its special crystalline arrangement, scPLA has superior thermal, mechanical and barrier properties than PLA^12,13^. Due to the chiral-complexation interaction between PLLA and PDLA chains, the melting point of scPLA is ~50 °C higher than PLLA or PDLA^8^.

scPLA can be prepared by either solution^14^ or melt blending^15^ of enantiomeric PLA in 1:1 ratio^16^. In the solution processing, scPLA can be prepared by precipitation or solution-cast method. Several other methods such as thin layer formation^10^, hydrolytic degradation etc. have been developed to form scPLA. Due to the presence of solvent, it is difficult to control over crystallization in case of solution processing. Also, these methods which involves organic solvents are not considered to be environmental friendly. In the melt-mixing process, two enantiomers of PLA are mixed by melting and cooled to form scPLA. In this process, homocrystals of PLA are also formed along with stereocomplex crystallites. It has been shown that the formation of stereocomplex crystallites is the result of relatively low molecular weight PLA chains. Another technique is developed by Purnama *et al*.^17^ to synthesize scPLA using supercritical fluids. Synthesis of stereoblock PLA, sintering and compression of enantiomeric PLA^18^, ternary stereocomplex formation via configurational molecular glue^19^ or use of fillers^20^ or biofillers, such as cellulose nano/micro crystals^21,22^, clays^23^, chitosan, hydroxyapatite^24^ etc. into the matrix of scPLA can be the possible routes for preventing the formation of homocrystals.

Among various biofillers, chitosan has been chosen for the present work due to its biodegradability, nontoxicity, biocompatibility along with antibacterial activity^25^. It is a naturally occurring polysaccharide which is obtained by deacetylation of chitin. Chitosan is soluble in acidic media due to the presence of free amino groups which get protonated in acidic environment. Chitosan has been used in many applications such as food industry, pulp and paper industry, wastewater treatment, agriculture etc.^26^. Due to its hydrophilic nature, its use in packaging and engineering applications is restricted.

Fortunati *et al*.^27^ have prepared biocomposites of PLA and chitosan by extrusion method and studied its properties. They found that chitosan and PLA are not compatible and do not form homogeneous dispersion of PLA-chitosan matrix. They also found that addition of chitosan led to significant decrease in the mechanical properties which proves that chitosan does not have the effect of reinforcement after adding into PLA matrix, rather it made PLA more fragile. Gartner *et al*.^28^ have coated PLA films with PLA/chitosan solution blend and tested the adhesion of PLA/chitosan blend on PLA films. Improvement in adhesion properties was achieved due to the use of methylenediphenyl 4, 4′-diisocyanate (MDI). They found that without MDI, chitosan had no contact with PLA films, which suggests non-compatibility of chitosan with PLA. Spiridon *et al*.^29^ have analyzed the effect of keratin on PLA/chitosan composite prepared using Brabender. They found that PLA degraded at a much faster rate in presence of chitosan and a decrease in mechanical properties was observed which suggests the need for further improvement in interfacial adhesion of chitosan and PLA. Torres-Huerta *et al*.^30^ have prepared PET/PLA/chitosan biocomposites using the extrusion method. They concluded that the presence of chitosan in the poly (ethylene terephthalate) (PET) matrix favors the degradation rate.

In this work, chemical modification of chitosan has been carried out during *in situ* condensation polymerization of lactic acid. After modification with low molecular weight oligomeric PLA, the *nano*-amphiphilic chitosan or modified chitosan (MCH) has been dispersed in PLA polymer granules which indicates the improved miscibility of chitosan in the polymer matrix. The biocomposite of PLLA, PDLA and MCH has been prepared using melt-blending process to form the scPLA and the effect of modified chitosan on structural, morphological, thermal and mechanical properties of scPLA has been studied.

## Materials and Methods

Poly-(L-lactic acid) was supplied by NatureWorks^®^ with trade name PLA-2003D. PDLA was synthesized via ring-opening polymerization (ROP) of bulk D-lactide in presence of tin octoate (Sigma Aldrich) as catalyst. The ROP was done in vacuum at 160 °C for 2 hours. Unreacted lactide in the synthesis process was removed by volatilization process for 2 hours at 110 °C. D-lactide was synthesized by two step process. D-lactic acid was purchased by Musashino Chemicals, China, which was oligomerized by removing the water content from the system at 150 °C followed by vaporization of the produced D-lactide via depolymerization of oligomer at 250 °C in presence of tin oxide (Sigma Aldrich) as catalyst. Produced D-lactide was purified by washing and recrystallization process followed by drying in vacuum at 60 °C. L-lactic acid was procured from Pioma Chemicals as brand name Purac PF-90. Chitosan (medium molecular weight) was purchased from Sigma Aldrich. All the materials were used as received. The properties of PLLA and PDLA are listed in Table 1.

**Table 1:** Properties of PLA used in the experiment.

Sr. No.	Material Name	T_g_ (°C)	T_m_ (°C)	M_w_ (kDa)	M_n_ (kDa)	PDI	Specific rotation [α]
01	PLLA (2003D)	57	157	183	100	1.8	−154°
02	PDLA	57	178	224	122	1.8	156°
03	Oligomer (MCH)	—	—	~3.0	~2.5	1.2	—

T_g_ and T_m_ were calculated using DSC second heating graph of specimen, the M_w_, M_n_ and PDI were calculated using GPC compared with standard polystyrene standard, the specific rotation [α] of the sample were measure using polarimeter at 25 °C with 1.0 gm/100 mL concentration in chloroform.

### Preparation of Modified Chitosan

Modified chitosan was synthesized using the procedure adopted from elsewhere^31^ wherein L-lactic acid and chitosan in the ratio of 3.33:1 (wt/wt %) were mixed appropriately in a round-bottom flask. The mixture was then left 12 hours for complete immersion of Lactic acid into chitosan. The immersed mixture was then kept under microwave (160 W) heating and inert atmosphere was maintained by purging nitrogen gas through RBF. Condensation polymerization reaction was carried out for lactic acid-grafted-chitosan or modified chitosan (MCH) preparation in microwave. The microwave was operated at 110 °C for 30 minutes. The moisture and other byproducts were removed during the reaction. After the reaction was complete, the microwave was turned off followed by the removal of inert atmosphere. Highly viscous product having dark brown color was obtained which was collected for further analysis and used for other applications.

### Preparation of biocomposite of PLLA/PDLA and MCH

The biocomposite of PLLA, PDLA and modified chitosan (MCH) was prepared by melt-blending in a twin screw extruder (HAAKE Minilab II) and molded using an injection molding machine (HAAKE Minijet Pro from Thermo Fischer Scientific, Germany). Firstly, all materials were dried in hot-air oven at 60 °C for 24 hours. PLLA and PDLA were mixed in 1:1 weight ratio and the desired percentage (0.5, 1.0 and 1.5%) of MCH was added into the mixture. The mixture was extruded in a twin screw extruder at 210 °C with screw speed 50 rpm. The produced blend was injection-molded with cylinder temperature 220 °C and mold temperature at 90 °C. The melt was injected with pressure of 700 bar and held for 5 seconds. The dumbbells (shown in Fig. S1, supplementary information) were removed from the mold after 10 seconds. The prepared biocomposite was named as scPLA-0.5%MCH, scPLA-1.0%MCH and scPLA-1.5%MCH for 0.5, 1.0 and 1.5% content of MCH whereas the blend of PLLA and PDLA was referred as scPLA.

### Apparatus

FTIR (Nicolet Magna-560) was used for the identification of possible intermolecular interaction between PLLA, PDLA and modified chitosan. The analysis was done by scanning the sample from 4500 cm^−1^ to 400 cm^−1^ with resolution of 4 cm^−1^ with accumulation of 64 scans. The samples were prepared as thin film by hot press.

Thermal properties of the biocomposite were measured by differential scanning calorimeter DSC 204F1 Phoenix from Netzsch (Germany) (pre-calibrated using Indium standards) equipped with CC300 N_2_ cooling system, under the nitrogen flow of 20 mL/min. The specimens were melted at 240 °C with a heating rate of 50 °C/min and kept at 240 °C for 5 min to remove the thermal history of the processing. After the removal of thermal history, the specimens were cooled to 30 °C with cooling rates of 2, 5, 10 and 15 °C/min and then heated to 240 °C with same heating rate as that of cooling.

Thermal degradation analysis of the biocomposite was done on TGA 4000 thermo gravimetric analyzer from Perkin Elmer. The samples (7–10 mg) were heated from 30 °C to 700 °C with a scan rate of 20 °C/min in 20 mL/min flow of nitrogen.

Shimadzu XRD-6000 X-ray diffractometer with copper K_α_ radiation (at 30 kV and 40 mA) and a monochromatized wavelength of 1.542 Å (0.1542 nm) was used to determine the crystal structure of samples. The scanning 2θ angles were ranging from 5° to 30° with a scanning rate of 2° per minute.

The degree of crystallinity of the specimens were calculated using Equations 1 and 2

1 $${X}_{c,sc}( \% )=\frac{{A}_{c,sc}}{({A}_{c,sc}+{A}_{a})}\times 100\,$$

2 $${X}_{c,hc}( \% )=\frac{{A}_{c,hc}}{({A}_{c,hc}+{A}_{a})}\times 100\,$$

where, *X*_*c*,*sc*_ and *X*_*c*,*hc*_ are the degree of crystallinity of stereocomplex and homo crystallites, respectively. *A*_*c*,*sc*_ and *A*_*c*,*hc*_ are the area for the stereocomplex, homo crystallites and *A*_*a*_ is the area of amorphous phase. The program for the heat treatment of sample is given in Fig. S2 (supplementary information).

The heat deflection temperature (HDT) of the specimen was estimated using the dynamic mechanical analyzer (DMA). The sample dimension was kept as 10 mm × 5 mm × 2 mm [Length (L) × Width (W) × Height (H)]. The HDT was measured using three point bending module. The force required for the analysis was calculated using Equation 3 mentioned in the ASTM- 648-07 standard as

3 $$F=\frac{2}{3}[\frac{\sigma ({H}^{2}\times W)}{L}]$$

where, σ is the stress on the specimen (0.455 MPa). The calculated force was 0.6067 N. The sample strain (ε) was calculated using Equation 4

4 $$\varepsilon =6\times \frac{{(Diflection)}_{ASTM}\times {(Thickness)}_{ASTM}}{{(Length)}_{ASTM}^{2}}$$

where deflection (ASTM), thickness (ASTM), and length (ASTM) mentioned in ASTM 648-07 standard were taken as 0.25 mm, 13 mm and 127 mm. The estimated strain i.e. 0.121% was used to calculate the deflection length (D) using Equation 5

5 $$D=\varepsilon \times \frac{{(length)}_{sample}^{2}}{6\times {(thickness)}_{sample}}$$

The calculated length of deflection was 10.08 µm. It means that the HDT estimated using DMA will be the temperature at which the sample deflects 10.08 µm with applied force of 0.6067 N at a heating rate 2 °C/min.

DMA analyses were performed using a dynamic mechanical analyzer DMA 242E Artemis from Netzsch GmbH. A three-point bending clamp was used at a frequency of 1 Hz and oscillating amplitude of 40 µm. The samples were heated from 30 °C to 100 °C at a heating rate of 3 °C/min in 100 mL/min nitrogen flow.

Tensile properties of the injection molded samples were measured using universal testing machine UTM from Kalpak, India. Five dumbbell shaped specimens were characterized for each composite and average of the data shown with standard deviation. The tests were performed at a crosshead speed of 5 mm/min using gauge length of 50 mm.

TEM analysis was done to determine the shape and size of modified chitosan and its distribution into the PLA matrix. The samples for analysis were prepared by placing a small drop of biocomposite solution on carbon coated TEM grid. The grid was kept at room temperature to evaporate and drain out the chloroform which left a thin layer of the biocomposite on grid. The TEM analysis was performed on the JEM 2100 from JEOL. The morphology of the fractured surface of scPLA and MCH biocomposite was characterized using Sigma FESEM from Zeiss USA operating at an acceleration voltage of 3–5 kV. The samples were obtained by fracturing the dumbbell in small pieces and stick on the stub using carbon adhesive tape and gold coated in sputtering unit before analysis.

## Results and Discussion

### Grafting analysis and stereocomplexation in PLA biocomposite

**Figure 1:**
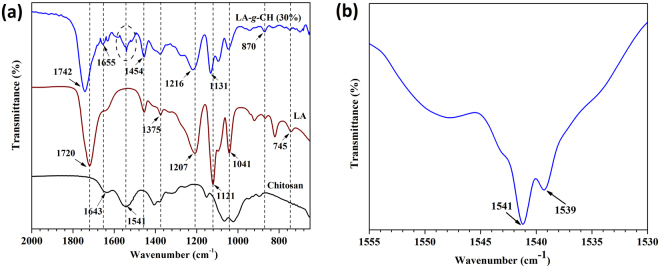
FTIR spectra of chitosan, lactic acid and LA-g-CH (MCH) in the range of 650 cm^−1^ to 2000 cm^−1^ (**a**) and FTIR spectra of LA-g-CH (MCH) in the range of 1530 cm^−1^ to 1555 cm^−1^ (**b**).

Analysis for grafting of lactic acid with chitosan has been carried out by FTIR analysis and the spectra is shown in Fig. 1. The regular peaks at 1541 cm^−1^ and 1655 cm^−1^ corresponding to strong N-H bending vibration of the secondary amides and strong -C=O stretching vibration of the primary amides respectively are observed for chitosan as well as for LA-g-CH (MCH). A characteristic peak at 1720 cm^−1^ is observed in lactic acid spectra which can be attributed to the presence of carboxyl group (-C=O) stretching vibrations. This peak is shifted towards the higher wavenumber at ~1742 cm^−1^ in case of LA-g-CH. It supports the grafting of lactic acid onto the chitosan backbone. The other regular peaks at 1454 cm^−1^, 1375 cm^−1^, 1121 cm^−1^, 1041 cm^−1^ and 870 cm^−1^ are detected in lactic acid spectra which correspond to -CH_3_ bending, -CH- bending, -C-O- stretching, -OH bending and -C-C- stretching respectively. These peaks are also observed in LA-g-CH sample but some of the peaks such as 1121 cm^−1^ and 1041 cm^−1^ are shifted towards higher wavenumber and located at 1131 cm^−1^ and 1045 cm^−1^. An additional new peak is observed in LA-g-CH sample at 1539 cm^−1^ witnessing the amide ester linkage (-OCONH-) between chitosan and lactic acid which leads to the grafting of growing lactic acid oligomers on the chitosan backbone.

**Figure 2:**
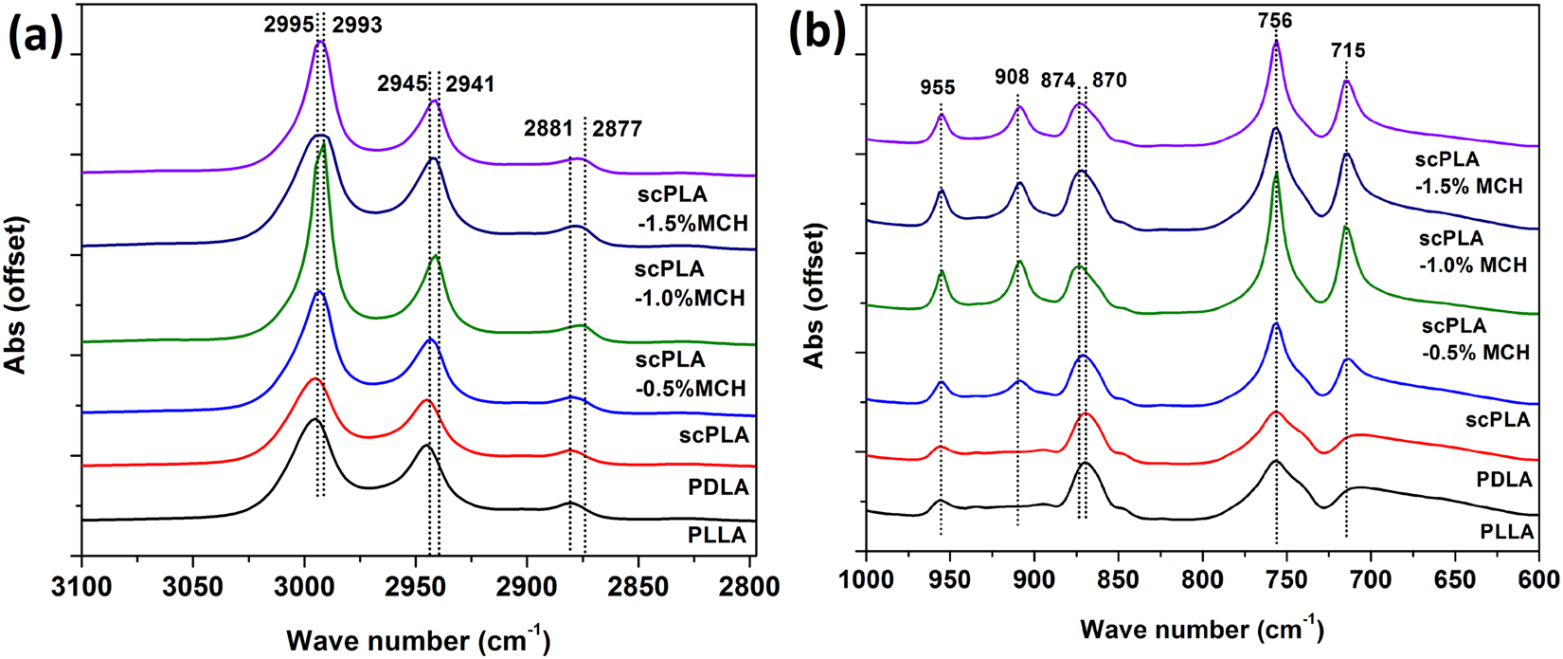
FTIR spectra of PLLA, PDLA, scPLA and MCH biocomposites in the range of 2800 cm^−1^ to 3100 cm^−1^ (**a**) and in the range of 600 cm^−1^ to 1000 cm^−1^ (**b**).

Further, the FTIR spectra of PLLA, PDLA, and its MCH biocomposite confirm the formation of stereocomplex crystallites. The principle peaks, related to stereocomplex crystallites formation, are summarized in Table 2. The IR band is shown in Fig. 2. It is known that the stereocomplex crystallites of PLA exhibit different FTIR spectra as compared to that of homo-crystallites. In Fig. 2b, a new peak is observed at 908 cm^−1^ is in case of stereocomplex and its MCH biocomposite. The new peak at 908 cm^−1^, is the characteristic band for the stereocomplex crystallites with 3_1_ helical confirmation. Its intensity is found to increase as with addition of MCH into the blend, suggesting more compact crystal packing. In Fig. 2a, as compared to the FTIR spectra of homo-PLLA crystallites, the stereocomplex spectra encountered peak shift for symmetrical (4 cm^−1^), and asymmetrical (2 cm^−1^) stretching of CH_3_ group and stretching of -CH group. Low frequency shift in case of stereocomplex crystallites suggests the interaction of the C=O and -CH which form weak hydrogen bond between the PLLA and PDLA chain in the crystal. High frequency shift in the band for stretching of C-COO from 870 to 874 cm^−1^, confirms the formation of the stereocomplex crystallites due to carbonyl group interaction with methyl group of opposite aligned anti enantiomeric PLA chain^32^.

**Table 2:** FTIR spectra assignment for scPLA-MCH biocomposites.

Sr. No.	Assignment	Wavenumber (cm^−1^)
01	Asymmetrical stretching of CH_3_	2995 → 2993
02	Symmetrical stretching of CH_3_	2945 → 2941
03	Stretching of CH	2881 → 2877
04	Rocking of CH_3_ and stretching of C-COO	955
05	Rocking of CH_3_ and stretching of C-COO and characteristic peak for the stereocomplex crystallite with 3_1_ helix confirmation	908
06	Stretching of C-COO	874 ← 870
07	Stretching and deformation of C-H, assigned to crystalline phase	756
08	Stretching and bending of C-H	715

**Figure 3:**
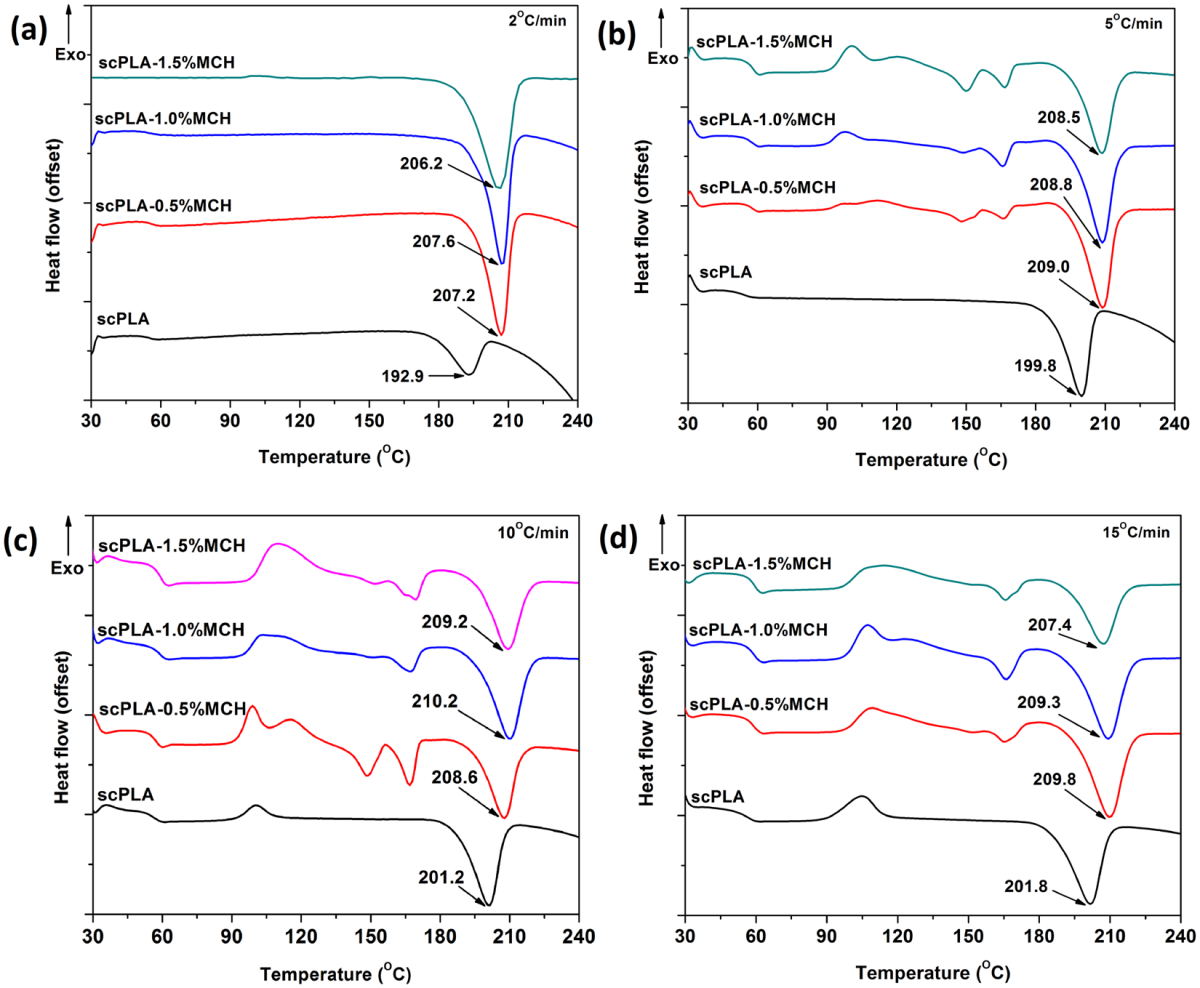
Second heating thermogram of scPLA and its MCH biocomposites with different scanning rate 2 °C/min (**a**), 5 °C/min (**b**), 10 °C/min (**c**) and 15 °C/min (**d**).

Melting behavior of the scPLA biocomposite has been characterized. The specimens were melted at 240 °C and subsequently cooled with several cooling rates i.e. 2, 5, 10, 15 °C/min. and again heated with different heating rate. Fig. 3 shows the DSC thermogram of scPLA and its MCH biocomposite. From Fig. 3, it can be seen that the heating/cooling rate has a significant effect on the stereocomplexation in PLA and it helps in suppressing the formation of the homocrystals. From Table S1 (supplementary information), it is found that the enthalpy of the homocrystals decreased with decrease in the heating rate. Increase in the melting temperature of the biocomposite as compared to neat scPLA is the result of addition of MCH into PLLA/PDLA matrix which significantly affects the formation of stereocomplex crystallites. When the heating rate was reduced from 15 °C/min to 2 °C/min, the exothermic peak area related to homo PLA disappeared. It can be observed from Fig. 3a that in case of PLLA/PDLA (scPLA), no lower melting peak was found. The melting temperature was found to increase from 192.9 °C for scPLA to 207.6 °C with addition of 1% MCH. Lower melting point of scPLA could be attributed to the formation of perfect homocrystals, whose endotherm is merged with that of imperfect stereocomplex crystallites, leading to the lowering of overall melting temperature of scPLA which is confirmed by the X-ray diffraction analysis in subsequent section. The addition of MCH is found to enhance the melting temperature which may be accounted to the presence of more oriented chain. Low molecular weight oligomeric PLA chains which are grafted on the surface of the chitosan may aid in the formation of the stereocomplex crystallites. The enthalpy of the melting of stereocomplex crystals is found to increase from 20.56 J/g to 39.35 J/g (Fig. S3, supplementary information) with the addition of 1% MCH in the composite.

### Effect of heat treatment on stereocomplexation

**Figure 4:**
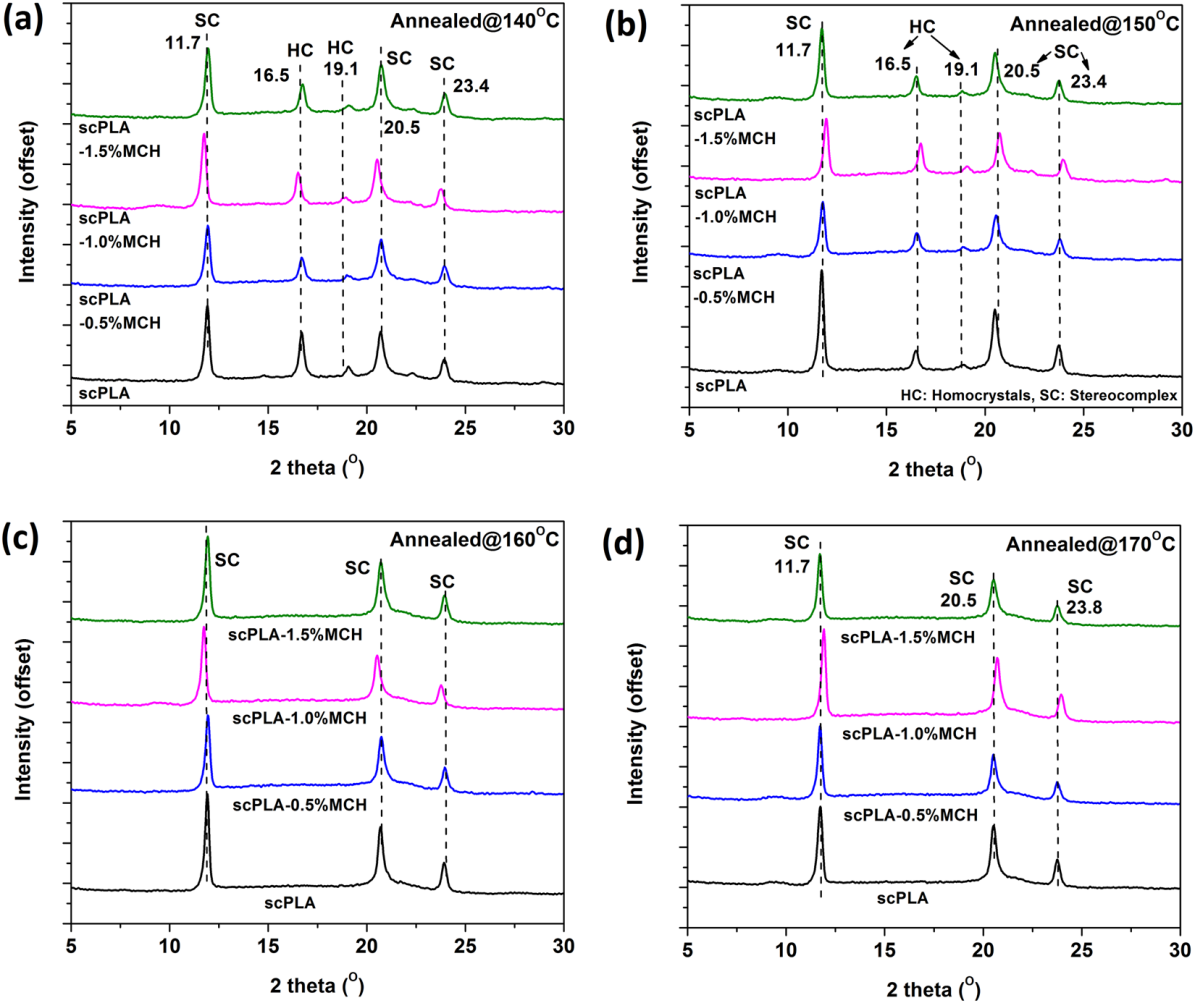
X-ray spectra of scPLA and its MCH biocomposites melt annealed at different temperatures 140 °C (**a**), 150 °C (**b**), 160 °C (**c**), 170 °C (**d**).

The scPLA and its MCH biocomposite specimens were melt-crystallized isothermally at different crystallization temperatures (140, 150, 160 and 170 °C for 2 hour) or non-isothermally at different cooling rates (2, 5, 10 and 15 °C/min) and subsequently, characterized by WAXD. It is known that the stereocomplex crystallites exist in the 3_1_-helical confirmation and are formed by parallel packing of L-lactide and D-lactide segments^10^. Homocrystals of PLLA or PDLA hold 10_3_ helical form. The diffraction peaks appearing at 17° and 19° are the characteristic peaks for the alpha form of the PLLA or PDLA which crystalized in a pseudo-orthorhombic unit cell^10^. The peaks in the diffraction pattern for stereocomplex crystallites appeared at 11°, 21° and 24°. Stereocomplex crystallites crystallized in a pseudo-trigonal unit cell^33^. The diffraction pattern for the isothermally crystallized specimens are shown in the Fig. 4. In case of isothermally crystallized specimen, it is found that the crystallization of samples at T_c_ of 150 °C or less than that gives peaks at ~17° and ~19° (Fig. 4a,b), which are the characteristic peaks for homo crystallites of PLA, as well as ~12°, ~21° and ~24°, which are the characteristic peaks for the stereocomplex crystallites of PLA. In case of specimens which have been crystallized at 160 °C or more than that, the peaks are observed at ~12°, 21°, and 24° (Fig. 4c,d)^34^. The results indicate that the stereocomplex crystallites are formed at all T_c_’s but no homocrystal appeared at temperature 160 °C or higher. The diffraction pattern in Fig. 4 shows that if the specimens are annealed at 150 °C or lower temperature, they tend to form stereocomplex crystallites along with homocrystals. However, if the specimens are annealed at 160 °C or more, formation of stereocomplex crystallites is observed while there is no evidence of homo crystals.

The calculated degree of crystallinity for stereocomplex and homo crystallites is shown in Fig. S4 (supplementary information). It can be seen that the homocrystals are found for specimens annealed at temperature 140–150 °C. However, in case of annealing temperature 160 °C and higher, there are no peaks corresponding to homocrystals. It is also confirmed that the degree of crystallinity for stereocomplex crystals does not vary significantly with T_c_. Only the peaks corresponding to homocrystals are diminished with annealing temperature. However, in case of scPLA, the melting temperature is found relatively lower with broader peak width which could be the result of the formation of non-uniform stereocomplex crystallites.

**Figure 5:**
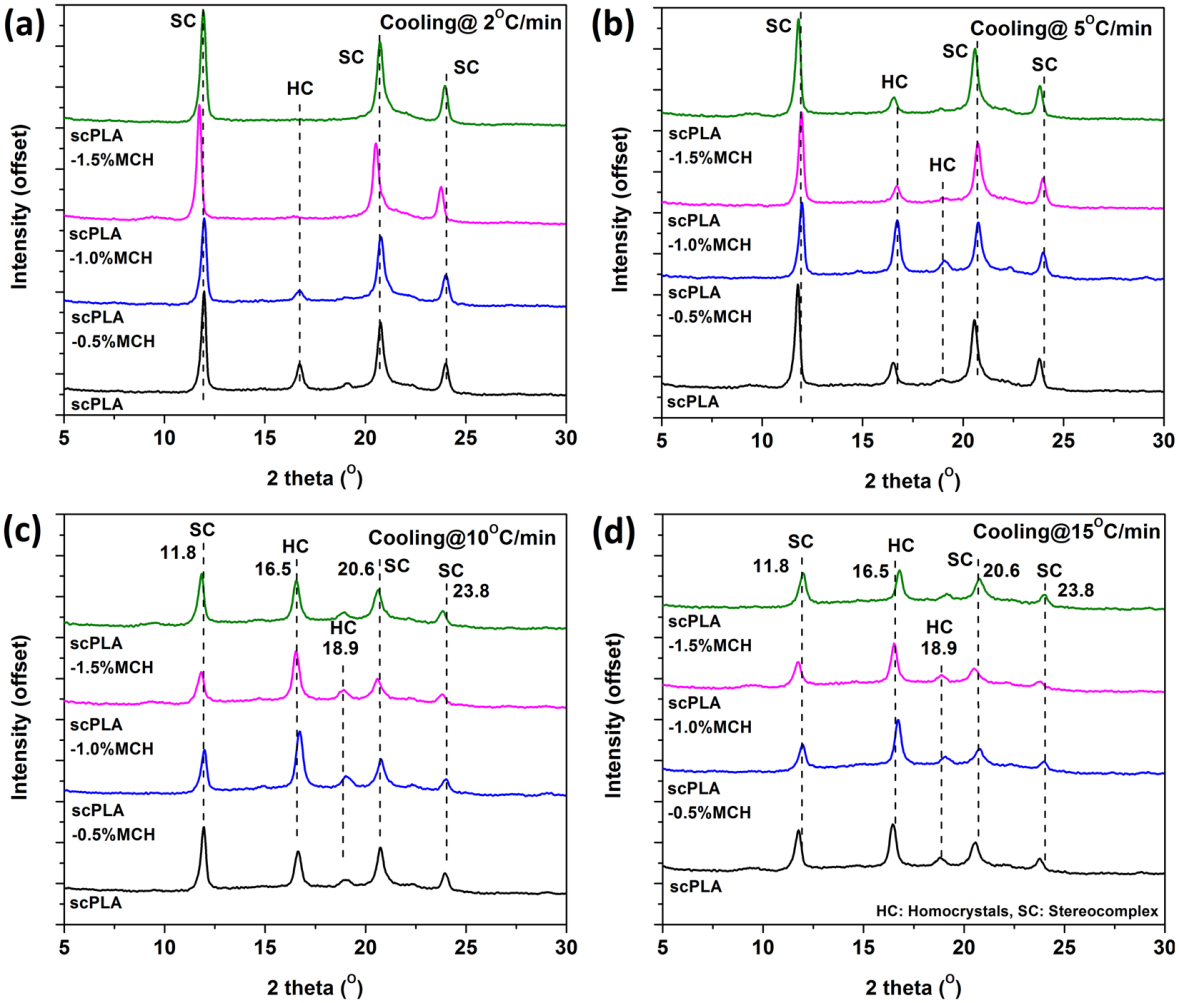
X-ray spectra of scPLA and its MCH biocomposites cooled at different cooling rate 2 °C/min (**a**), 5 °C/min (**b**), 10 °C/min (**c**) and 15 °C/min (**d**).

**Figure 6:**
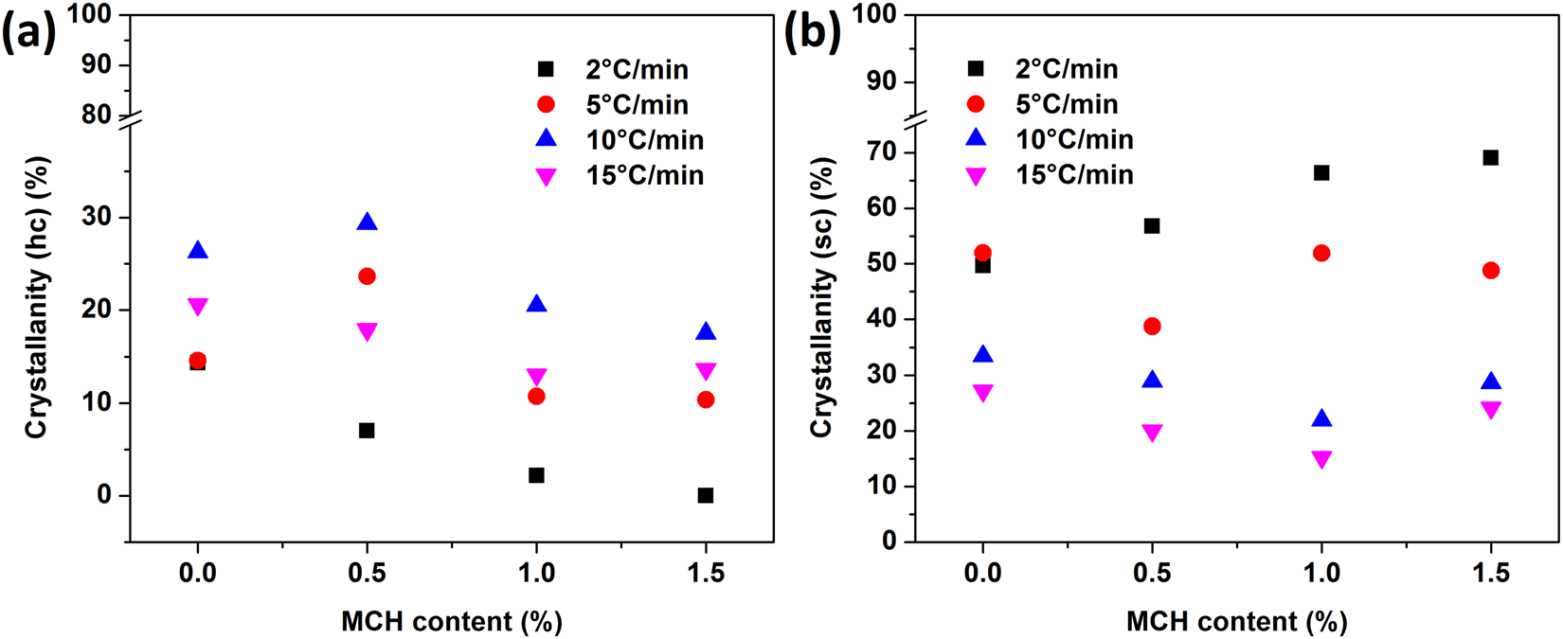
Crystallinity of scPLA and its MCH biocomposites after non-isothermal crystallization for homocrystals (**a**) and stereocomplex (**b**).

In case of the specimens crystallized non-isothermally, it is found that MCH content and cooling rate have a significant role in the formation of stereocomplex crystals and also inhibit the formation of homocrystals at lower cooling rates. From Fig. 5, it is found that the content of MCH helps in the formation of stereocomplex crystals. At a cooling rate 2 °C/min, the peaks related to homocrystals disappeared and only peaks corresponding to stereocomplex crystals were present. The degree of crystallinity for non-isothermally crystallized specimens are shown in Fig. 6. As the content of MCH increases, the total crystallinity also increases. The degree of crystallinity for stereocomplex crystallites is increased to ~70% for 1.5% MCH content and no evidence is found for the homocrystals. With increase in the cooling rate, the total crystallinity decreases.

In pristine scPLA, both stereocomplex and homocrystals are grown, which could lead to the decrease in the properties. More interestingly, only addition of MCH into the matrix would allow the growth of stereocomplex crystallites when melt-cooled with low cooling rate. Melt cooling of the specimen with low cooling rate gives enough time to the chains to orient in ordered structure, as depicted in Fig. S5 (supplementary information) and Fig. S6 (supplementary information) illustrates bonding between chitosan and PLA chains and the formation of stereocomplex crystallites due to intramolecular bonding between carbonyl carbon and methyl group of PLA species. The percentage of stereocomplex crystals increases with increase in the MCH content. This could possibly result from the MCH providing extra molecular surface area through small grafted PLLA chains which gives large interfacial area along with plasticizing effect. It is already identified that the stereocomplex crystallites are formed easily with low molecular weight PLA chains as compared to that of high molecular weight PLA chains^14^.

### Gel formation due to stereocomplexation

It is known that the stereocomplex crystallites of PLA do not dissolve in chloroform, but form the gels in the solvent. The gel content (gel%) is measured as per the literature^35^ given as

6 $$gel \% =\frac{{W}_{gel}}{{W}_{i}}\times 100\,$$

where, W_i_ and W_gel_ are the initial mass of biocomposite and mass of dried gel after washing with chloroform followed by vacuum drying, respectively.

**Figure 7:**
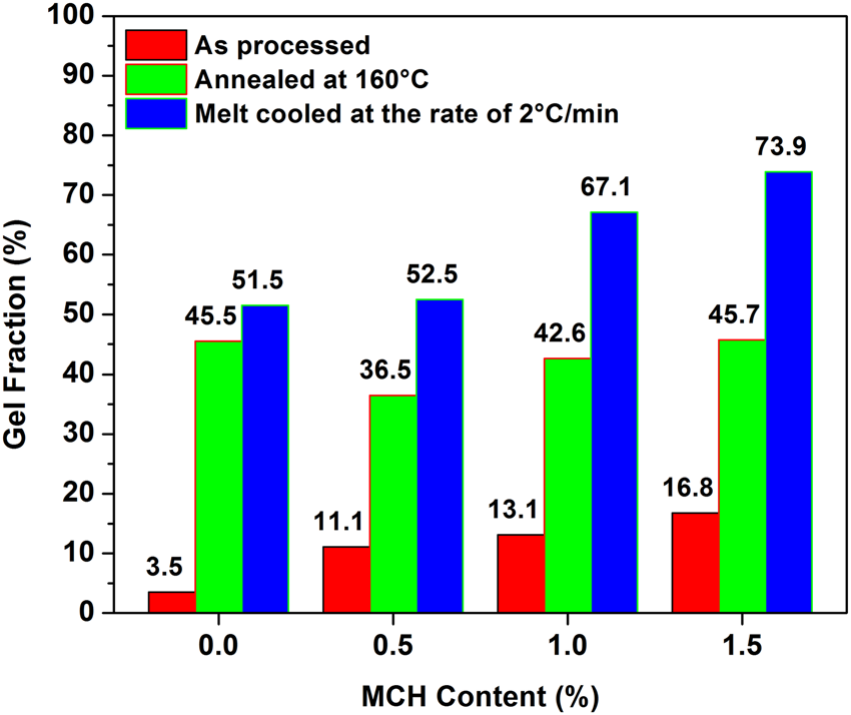
Gel fraction of scPLA and MCH biocomposites after washing with chloroform.

Figure 7 shows the gel fraction of the PLA MCH biocomposite with respect to the content of MCH. It is evident that the annealing and melt cooling has a significant effect on the formation of stereocomplex crystallites. In case of prepared specimens, the gel fraction of scPLA was ca. 3% which increased to more than 15%. It confirms the influence of MCH on stereocomplexation of PLA. The gel fraction is increased after annealing the specimen at 160 °C. The fraction is approximately 40% for all the composites. A significant enhancement is noted in case of melt cooling with a cooling rate of 2 °C/min where the gel fraction increased to more than 70%. Cooling at 2 °C/min provides enough time for the molecules to be arranged. Presence of the MCH increased the molecular surface area for the formation of the stereocomplex crystallites. It is found that the rate of formation of stereocomplex crystallites is relatively low thus in order to produce large quantity of biocomposite, additional heating cooling system may be required with ordinary processing units.

### Viscoelastic property and heat deflection temperature

**Figure 8:**
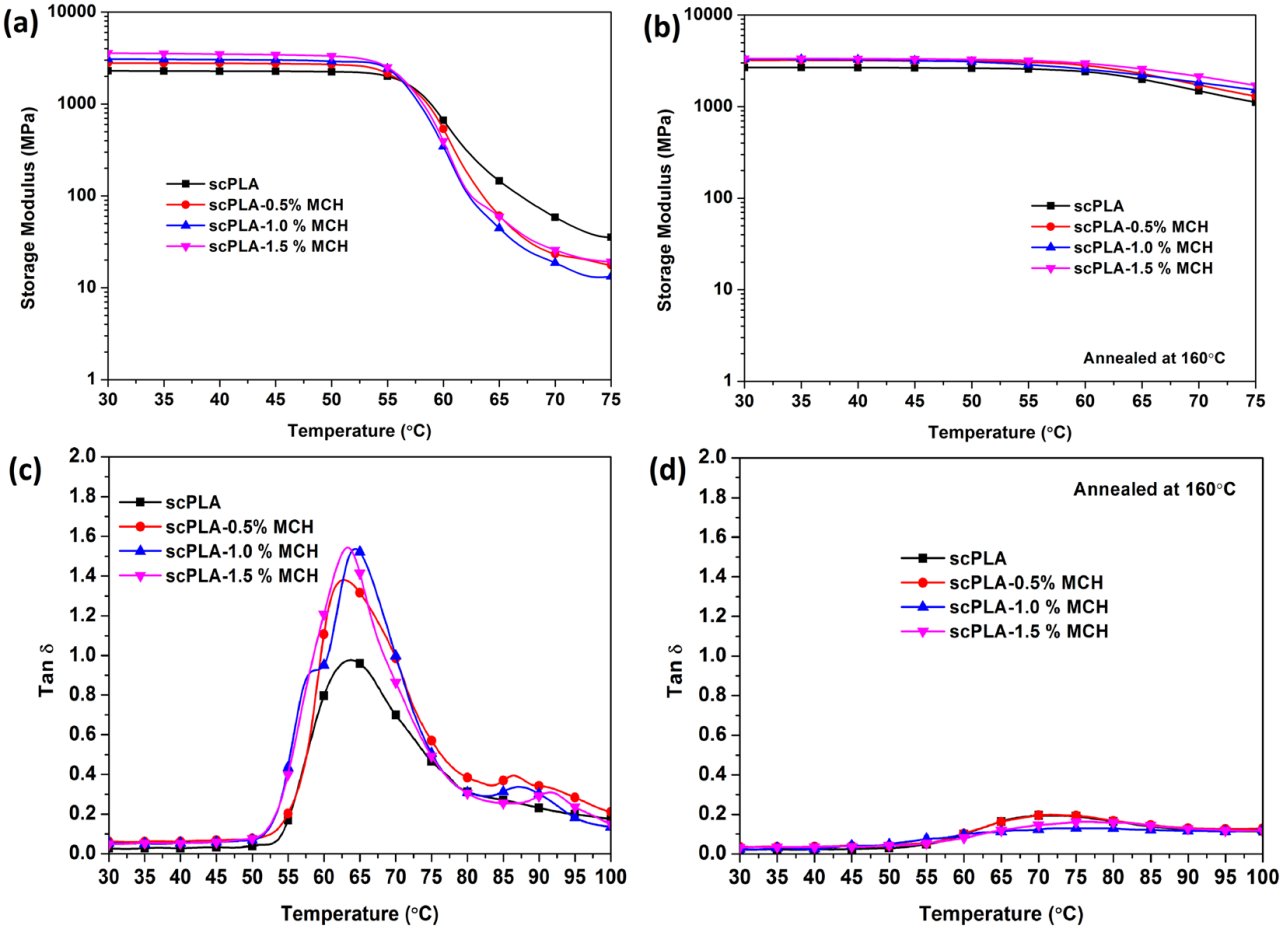
Storage modulus before (**a**) and after (**b**) annealing at 160 °C and tan delta before (**c**) and after (**d**) annealing at 160 °C of scPLA or its composites with varying content of MCH.

The viscoelastic properties of scPLA with MCH biocomposite were studied before and after annealing using dynamic mechanical analyzer. Figure 8 shows the storage modulus and tan delta of the scPLA with varying content of MCH before annealing (Fig. 8a,c) and after annealing at 160 °C (Fig. 8b,d). The storage modulus of scPLA was found to be 2295 MPa which increased to 3580 MPa after the addition of MCH to the scPLA matrix. Below glass transition temperature (T_g_), the biocomposite is in glassy state and no change is observed in the shape of the molecular chains due to the frozen chain segmental motion. The storage modulus of the biocomposite is drastically reduced after 57 °C, which is the glassy region of the biocomposite. Above glass transition temperature, the region is called the glass-rubber transition or softening region where the micro-Brownian motion of the polymer chains begin due to the presence of free volume beside the chains which permits the chain segments to rotate. The sudden drop of the storage modulus in the glass transition region is due to the initiation of the micro Brownian motion in the molecular chain. As the temperature increases, the average distance between neighboring molecules also increases which leads to the enhanced free volume. After annealing the sample at 160 °C, decrease in the storage modulus is highly reduced after 57 °C which suggests that the material became crystalline and stiff and does not display the glass transition. A crystallized chain segment can absorb or store much more energy for the given condition than free chain segments. At this point, the segmental motion of the chain is restricted due to the decreased free volume. The increase in the degree of crystallinity, due to annealing, reduces the free volume of the matrix and freezes the molecular motion. The tan delta, which is the measure of internal friction of the material at a particular condition was found to be more than 1 for the biocomposite without annealing. It confirms that the loss modulus is higher than the storage modulus of the biocomposite. It suggests that the viscous behavior of the biocomposite dominates over the elastic behavior. After annealing the samples at 160 °C, the tan delta reduced drastically to less than one. It suggests that the biocomposite is elastic, crystalline and stiff in nature. The enhanced moduli of the sPLA with MCH content may be the result of rigid interphase formed in the vicinity of crystalline stereocomplexes. The PLA chains attached with the chitosan molecules which acts as extended molecular surfaces and interact with the other PLA species present in the system and form the stereocomplex crystallites. The formation of stereocomplex crystallites leads to the development of interphase domains due to presence of chitosan chains in the vicinity of the stereocomplex crystallites. The crystalline nature of the biocomposite and the presence of chitosan into the matrix may also be responsible for the improved heat deflection temperature.

**Figure 9:**
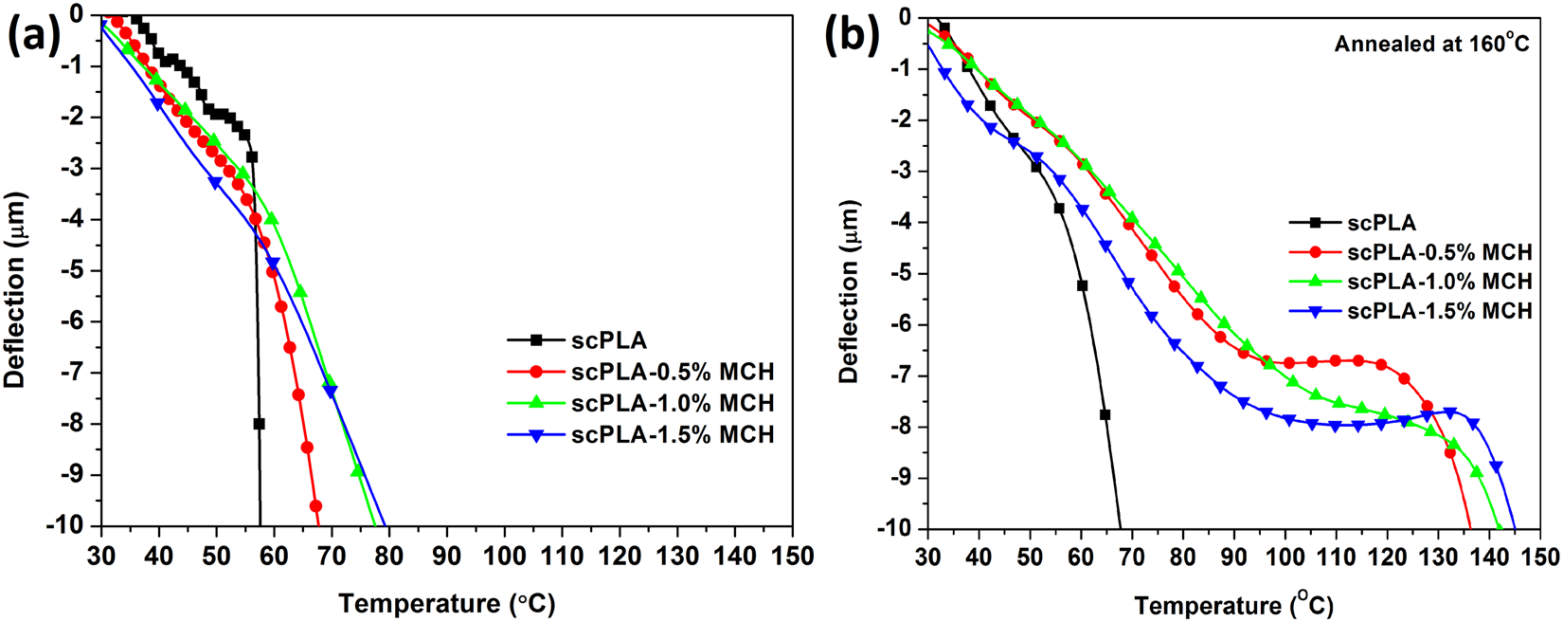
Heat deflection of scPLA or its composites with varying amount of MCH with change in temperature before annealing (**a**) and after annealing at 160 °C (**b**).

Heat deflection temperature is estimated by observing the temperature at which the polymer deflects under a controlled stress using dynamic mechanical analyzer. Fig. 9 shows the deflection length with respect to the temperature for scPLA with varying amount of MCH before (Fig. 9a**)** and after (Fig. 9b**)** annealing at 160 °C. A significant improvement is noticed in heat deflection temperature (HDT) when MCH is added to scPLA. The HDT of scPLA, as prepared, is estimated as ~58 °C which is improved to ~80 °C after addition of the 1.5% MCH. After annealing at 160 °C for 2 hours, HDT of scPLA enhanced to ~68 °C and improved to 145 °C in case of 1.5% MCH. The reinforcement of the modified chitosan in the PLLA/PDLA matrix leads to enhancement in the HDT. The network formation between PLA matrix and modified chitosan increases the HDT of biocomposite. It is known that the addition of the fibrous component into the polymer matrix enhances the HDT^36^. The chitosan microspheres reinforced into the scPLA interact with the PLLA and PDLA chains and restrict the movement of polymer chains by reducing the segmental freedom of movement. The low molecular weight chains grafted on the surface of the chitosan interact with other PLA species in the matrix forming the stereocomplex crystallites. Presence of chitosan and formation of the stereocomplex crystallites reduce the chain slippage which ultimately leads to the improvement in the HDT of the biocomposite, which could also be confirmed by the lower value of tan delta in DMA analysis. In other words, during the formation of stereocomplex crystallites, either isothermally or non-isothermally, MCH act as plasticizing agent and facilitate the high molecular weight PLLA and PDLA chains movement and its interaction with each other thus formation of stereocomplex crystallites. During the formation of stereocomplex crystallites, the low molecular weight PLLA chains attached to the chitosan molecule may engaged with high molecular weight enantiomeric PLA chains. Due to formation of stereocomplex crystallites, the chitosan dispersed in the polymer matrix become rigid entity and strict the segmental movement of PLLA and PDLA which make a firm complex structure thus leads to enhancement in the heat deflection temperature (HDT).

It is understood that addition of MCH into the PLA matrix enhances the crystallizability of the biocomposite and it is also known that the crystallization process is governed by the nucleation phenomenon such as free energy of nucleation (ΔG^*^). The nucleation constant (K_g_) was calculated using Lauritzen and Hoffmann Equation S1 (supporting information) by replacing spherulite growth rate with half time of crystallization (t_1/2_) and K_g_ value was used to measure ΔG^*^. The measured values were condensed in the Table S2 (supporting information). It can be seen from the Fig. S9 (supporting information) that the t_1/2_ values was reduced as the MCH content increased in the matrix which suggest that the presence of MCH enhance the crystallizability of the biocomposites. Nucleation constant enhanced from 6.3 × 10^5^ (K^2^) for sPLA to 7.7 × 10^5^ (K^2^) sPLA-1.5%MCH which suggests that the presence of MCH contribute into the chain mobility. The calculated value of product of lateral and fold free energies is higher for sPLA-MCH (624.0 erg^2^.cm^−4^) in comparison to sPLA (507.7 erg^2^.cm^−4^). Enhancement in the free energy confirms that the modified chitosan is not contributed as nucleating agent but provided the extended molecular surface of PLLA to interact with PDLA. This interaction may be responsible for the uniform nucleation which require higher energy of nucleation as confirmed by the increased nucleation energy (8.7 × 10^−13^ erg) in comparison to sPLA (7.1×10^−13^ erg).

**Figure 10:**
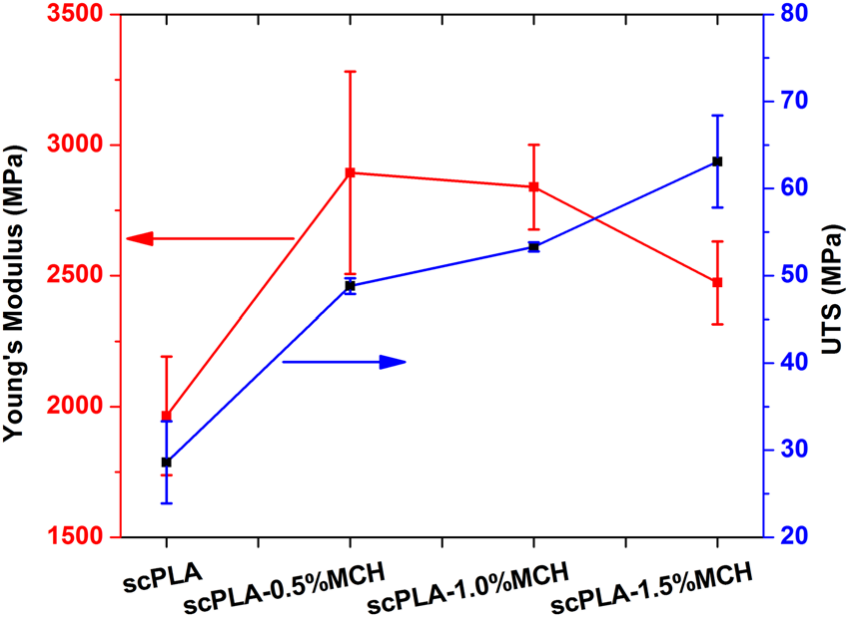
Ultimate tensile strength (UTS) and Young’s modulus of scPLA or its MCH biocomposites.

Ultimate tensile strength and Young’s modulus of scPLA and MCH biocomposite was also measured and shown in Fig. 10. It can be seen that the UTS improved from ~29 MPa for scPLA to ~63 MPa for scPLA with 1.5% MCH content. In case of Young’s modulus, it increased from ~1.9 GPa for scPLA to 2.8 GPa for 1.0% MCH content. This improvement in the biocomposite may be found due to the formation of stereocomplex crystallites which is formed due to the presence of MCH.

### Distribution of MCH and morphology of biocomposite

**Figure 11:**
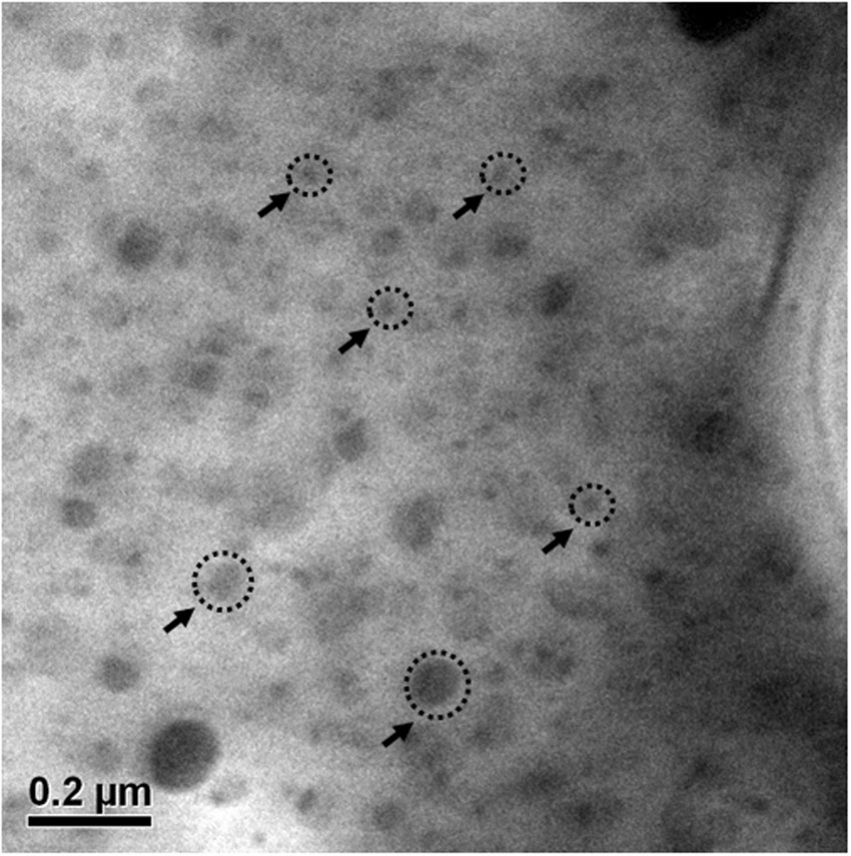
TEM image of the modified chitosan hanging in the matrix of PLA.

The distribution of MCH is analyzed using TEM which showed that three dimensional, spherical MCH particles were uniformly dispersed in the PLA matrix as shown in Fig. 11. The surface morphology of the biocomposite films showed two phases; one being the continuous polymer matrix and the other being discontinuous MCH phase in the form of spherical particles which showed a core-shell like structure. The shell of spheres is hydrophobic while the core is hydrophilic in nature. The dispersion of PLA and MCH is based on hydrophobic-hydrophilic interaction which creates only the spherical shape because it is the most favorable shape due to the lowest surface energy when repulsion forces act from all sides on the MCH particles.

The morphology of the fractured surface of PLA matrix and MCH biocomposite is studied by FESEM as shown in Fig. S7 **(**supplementary information). From the figure, it can be clearly seen that the smoothness of the polymer is disturbed due to the presence of MCH and where the surface turned rough as compared to that of neat polymer. Nano/micron sized nodes of MCH are distributed all over the surface which increased the molecular surface area responsible for the formation of stereocomplex crystallites.

### Thermal degradation behavior

It is well known that the presence of low-molecular-weight PLA chain may affect the degradation behavior and may reduce the degradation temperature drastically due to the self-catalytic degradation mechanism. The TGA analysis of the specimens are shown in the Fig. S8 (supplementary information). From the figure, it is found that there is no weight loss at lower temperature. For all the specimens, minor improvement is found in the onset degradation temperature. The onset degradation temperature of scPLA is found to be 348.2 °C and little improvement of ~3 °C is found for 1% MCH content. The temperature of maximum rate of degradation is found to be 380.3 °C for scPLA and 377.9 °C for 1% MCH content. The thermal analysis confirms that the presence of oligomeric PLA grafted on the chitosan in the biocomposite matrix does not affect the degradation temperature of the scPLA. The low-molecular-weight PLA engaged in the formation of the stereocomplex crystallites holds chitosan with in the matrix in biocomposite by providing the increased molecular surface area for PLLA and PDLA chain interaction.

## Conclusions

Chemical modification of chitosan has been successfully carried out and the biocomposite of high molecular weight PLLA/PDLA and MCH has been prepared using melt extrusion process. The prepared biocomposite is found to have the melting temperature more than 207 °C with ~40 J/g enthalpy in case of 1% MCH content. Formation of the stereocomplex crystallites in the biocomposite has been proved by using FTIR and XRD analysis. It has been found that the heat treatment had a significant effect on formation of the stereocomplex crystallites. When annealed at or more than 160 °C, no homocrystals were formed, however the degree of crystallinity is found to be ~40%. In case of nonisothermal cooling of biocomposite with the rate of 2 °C/min, the degree of crystallinity increased to ~70% for 1.5% MCH content and no homocrystallites were found. The gel fraction of the biocomposite after and before annealing also confirm the formation of stereocomplex crystallites. Due to the presence of MCH, being responsible for the enhancement in the formation of stereocomplex crystallites into PLA matrix, the heat deflection temperature has been found to improve from ~70 °C for scPLA to 145 °C for scPLA with 1.5%MCH. Due to the formation of stereocomplex crystallites and presence of chitosan, the ultimate tensile strength is found to enhance from ~29 to ~63 MPa and the modulus from ~1.9 to ~2.8 GPa. Thermal gravimetric analysis also confirms that there is no deteriorating thermal effect due to the presence of MCH into the polymer matrix.

## Electronic supplementary material

Supplementary Information
