# Supplementary material for: Effects of Amphiphilic Chitosan on Stereocomplexation and Properties of Poly(lactic acid) Nano-biocomposite
Source: Sci Rep. 2018 Mar 12;8:4351. doi: 10.1038/s41598-018-22281-1 (PMC5847540; doi:10.1038/s41598-018-22281-1)
Supplement: Supplementary file 1 — Supplementary Information [file 41598_2018_22281_MOESM1_ESM.doc]

**Supplementary Information**

**Effects of Amphiphilic Chitosan on Stereocomplexation and Properties of Poly(lactic acid) Nano-biocomposite**

Arvind Gupta^1^, Akhilesh Kumar Pal^1^, Eamor M. Woo^2^, and Vimal Katiyar^1*^

^1^Department of Chemical Engineering, Indian Institute of Technology Guwahati,-781039, Assam, India

^2^Department of Chemical Engineering, National Cheng Kung University, Tainan 701-01, Taiwan

*Corresponding Author: [vkatiyar@iitg.ernet.in](mailto:vkatiyar@iitg.ernet.in)

**Contents:**

**Figure S1**: Processed dumbbells of scPLA with varying amount of MCH

**Figure S2**: Thermal treatment program for X-ray diffraction analysis

**Figure S3**: Comparison of melting temperature (a) and melting enthalpy (b) with respect to MCH content at different heating rate

**Figure S4**: Degree of crystallinity of scPLA and its MCH biocomposite after isothermal crystallization for homocrystals (a) and stereocomplex (b)

**Figure S5**: Schematic for melt blending of PLLA/PDLA (I), Chitosan modification (II) and heat treatment of scPLA-MCH biocomposite (III)

**Figure S6**: Schematic of bonding between chitosan and PLA chains and the formation of stereocomplex crystallites due to intramolecular bonding between carbonyl carbon and methyl group of PLA species

**Figure S7**: FESEM images of fractured surface of scPLA (a) and scPLA-MCH biocomposite (b) scale 2 µm

**Figure S8**: Thermal gravimetric analysis of scPLA with varying percentage of MCH (a), the derivative weight corresponds to the temperature (b)

**Figure S9:** Relative crystallinity and t_1/2_ of sPLA and sPLA-MCH biocomposite at 120°C, 140°C and 160°C.

**Table S1**: Thermal properties of scPLA and biocomposites with various amount of MCH

**Table S2:** Thermodynamic and kinetic parameters for the isothermal crystallization of sPLA‑MCH biocomposite.


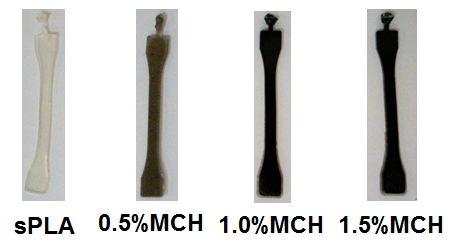


**scPLA**

**Figure S1:** Processed dumbbells of scPLA with varying amount of MCH

**
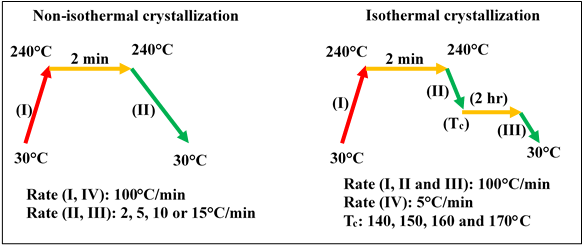
**

**Figure S2**: Thermal treatment program for X-ray diffraction analysis


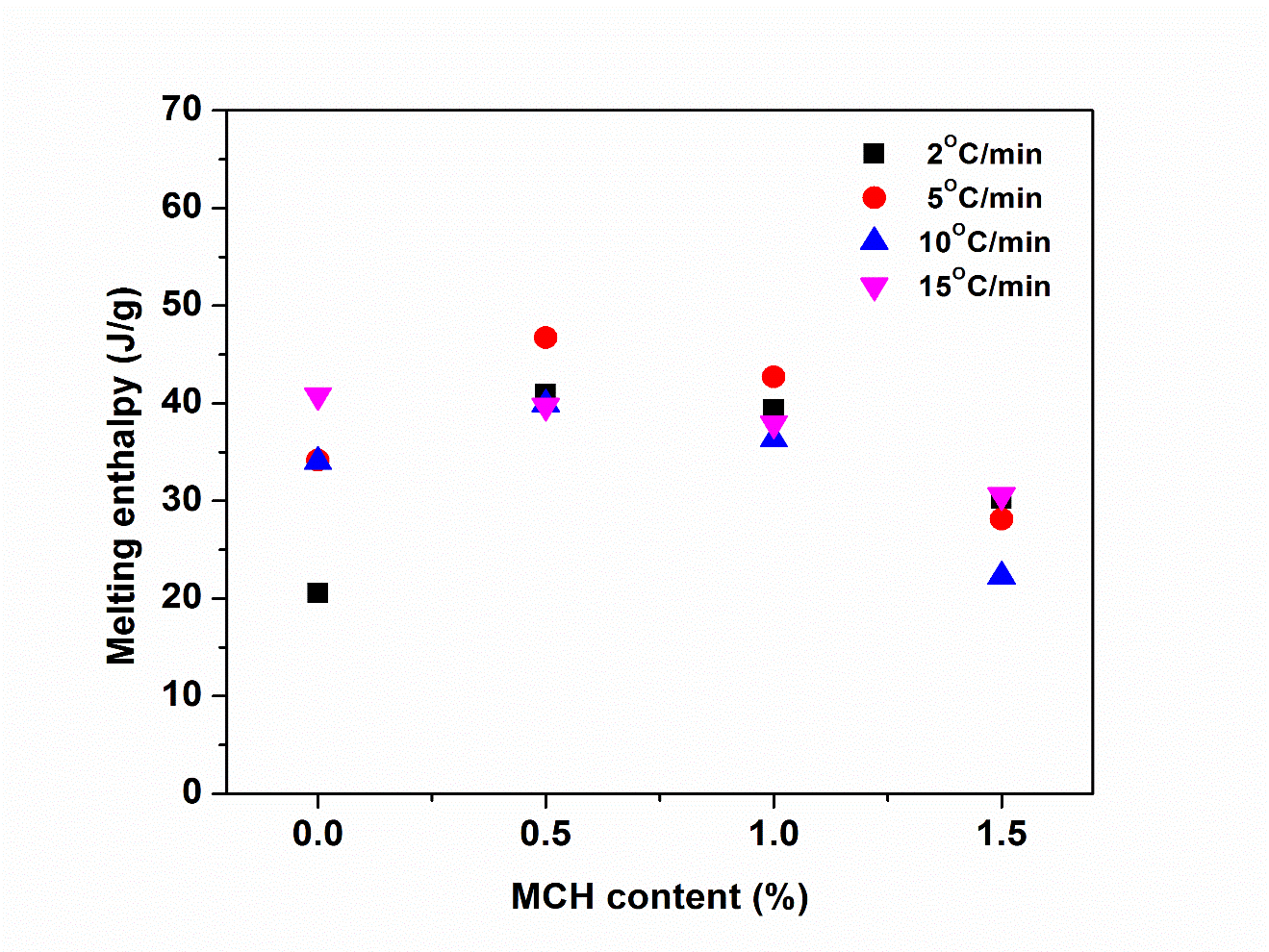

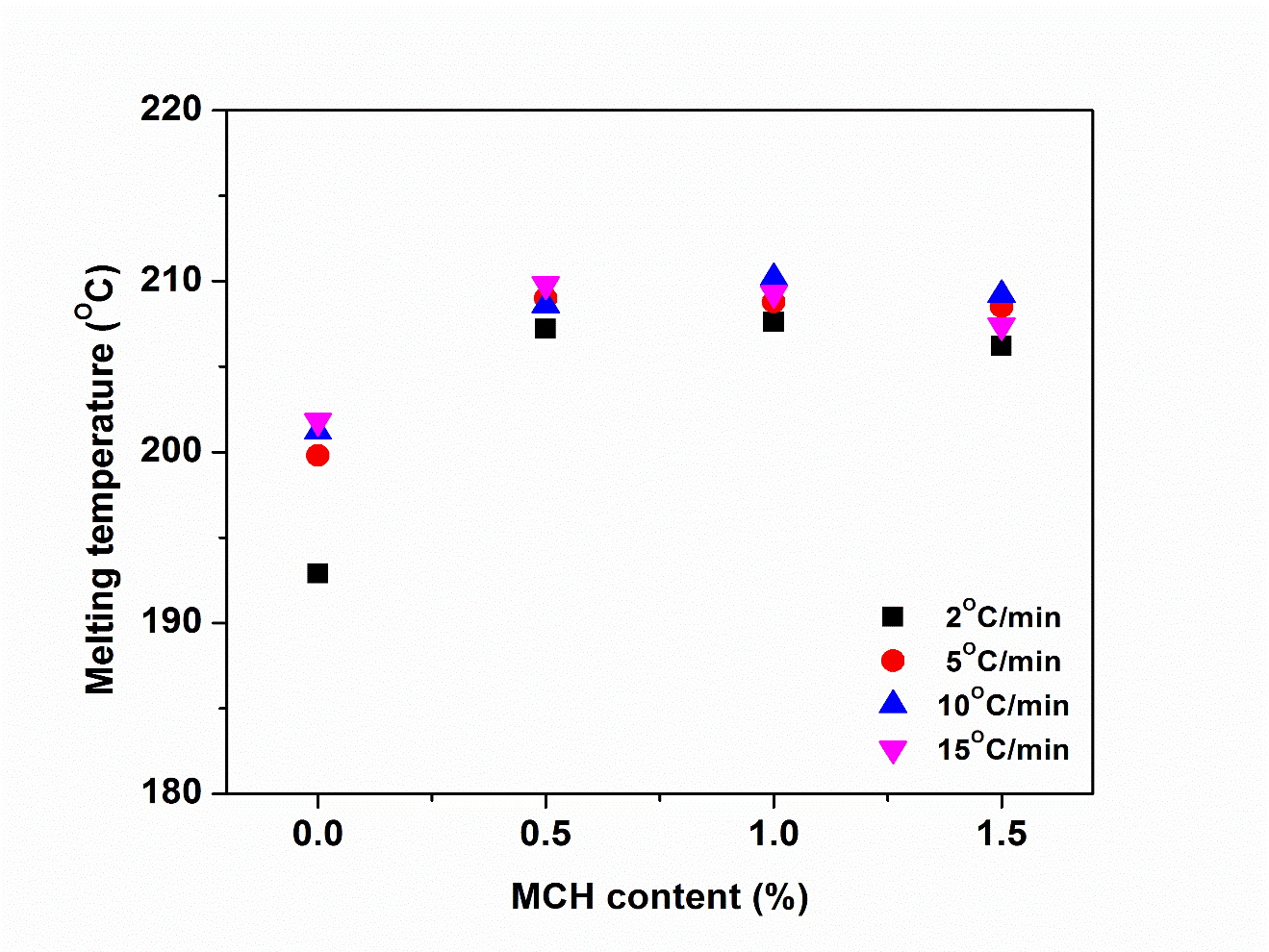


**(a)**

**(b)**

**Figure S3:** Comparison of melting temperature (a) and melting enthalpy (b) with respect to MCH content at different heating rate


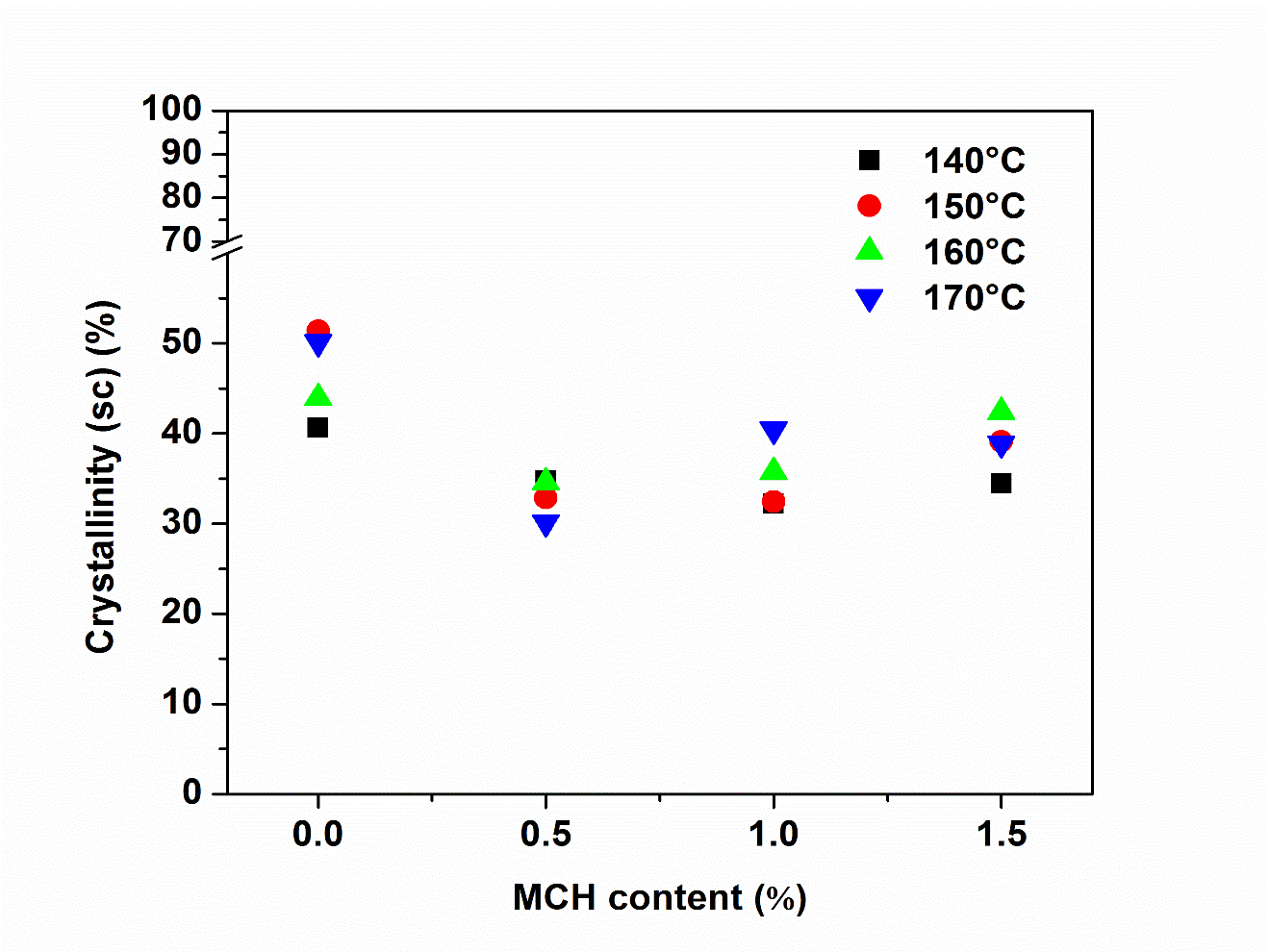


**(b)**


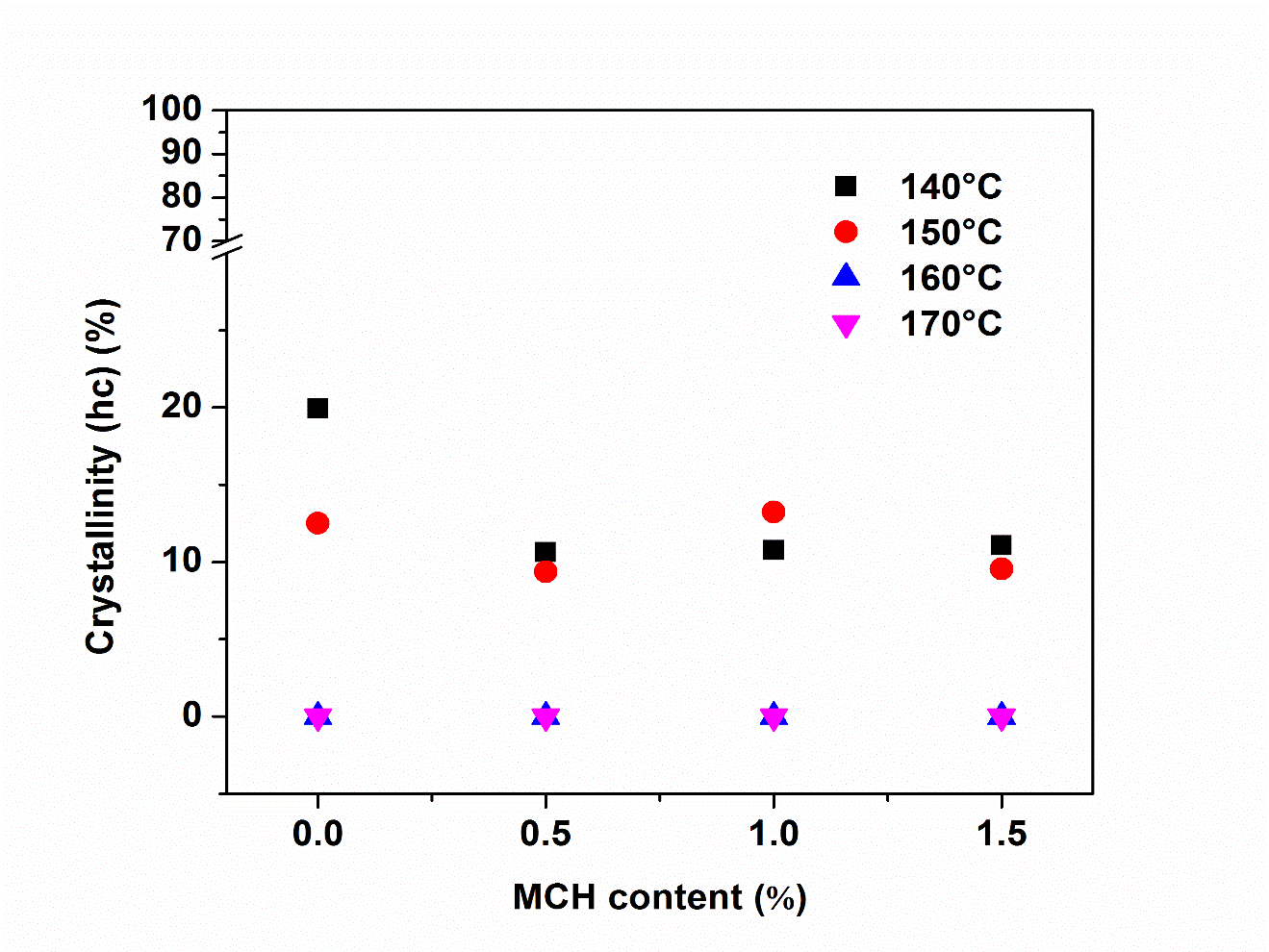


**(a)**

**Figure S4**: Degree of crystallinity of scPLA and its MCH biocomposite after isothermal crystallization for homocrystals (a) and stereocomplex (b)


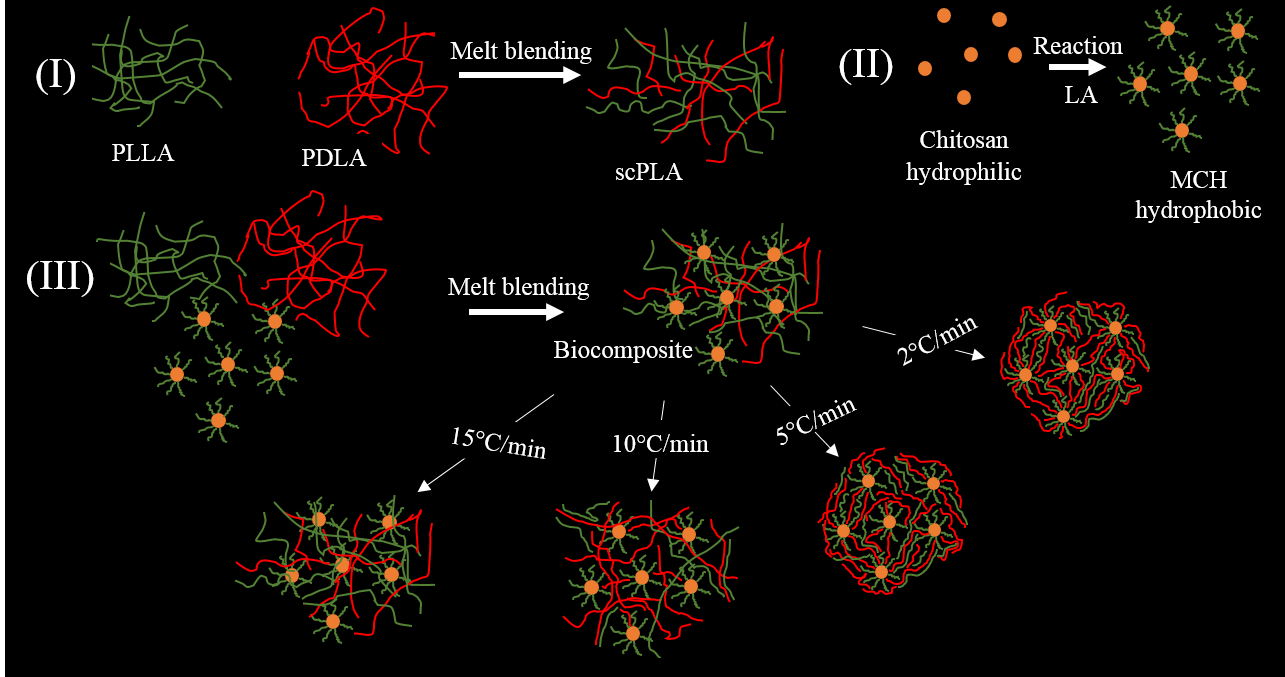


**Figure S5**: Schematic for melt blending of PLLA/PDLA (I), Chitosan modification (II) and heat treatment of scPLA-MCH biocomposite (III)


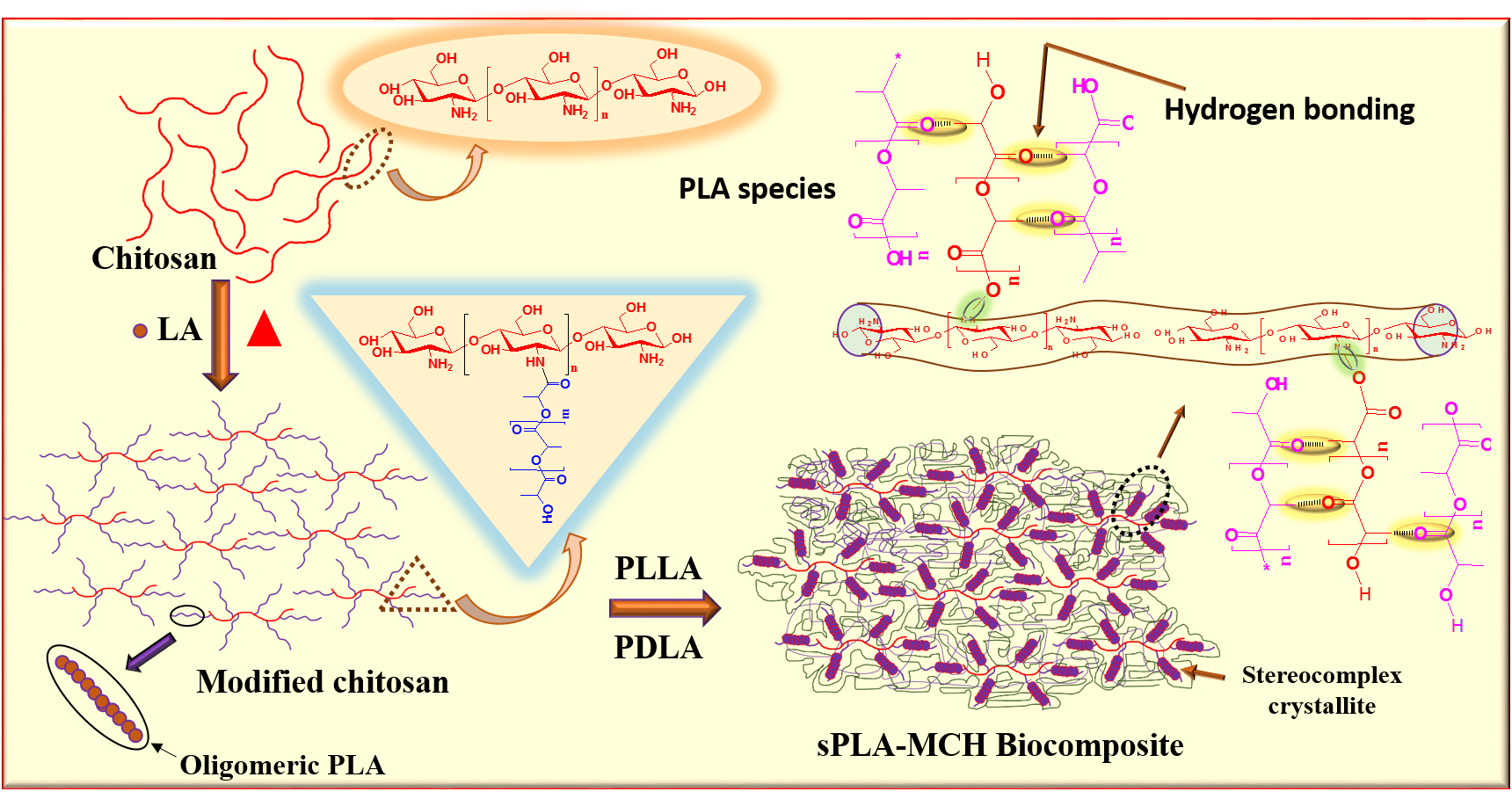


**Figure S6**: Schematic of bonding between chitosan and PLA chains and the formation of stereocomplex crystallites due to intramolecular bonding between carbonyl carbon and methyl group of PLA species


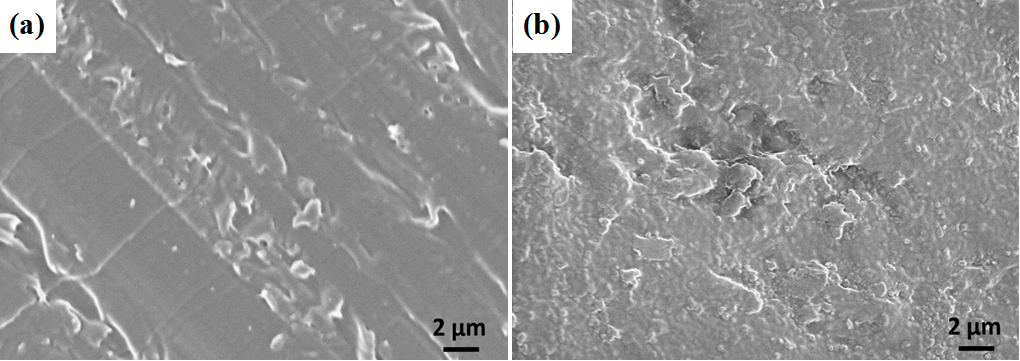


**Figure S7**: FESEM images of fractured surface of scPLA (a) and scPLA-MCH biocomposite (b) scale 2 µm


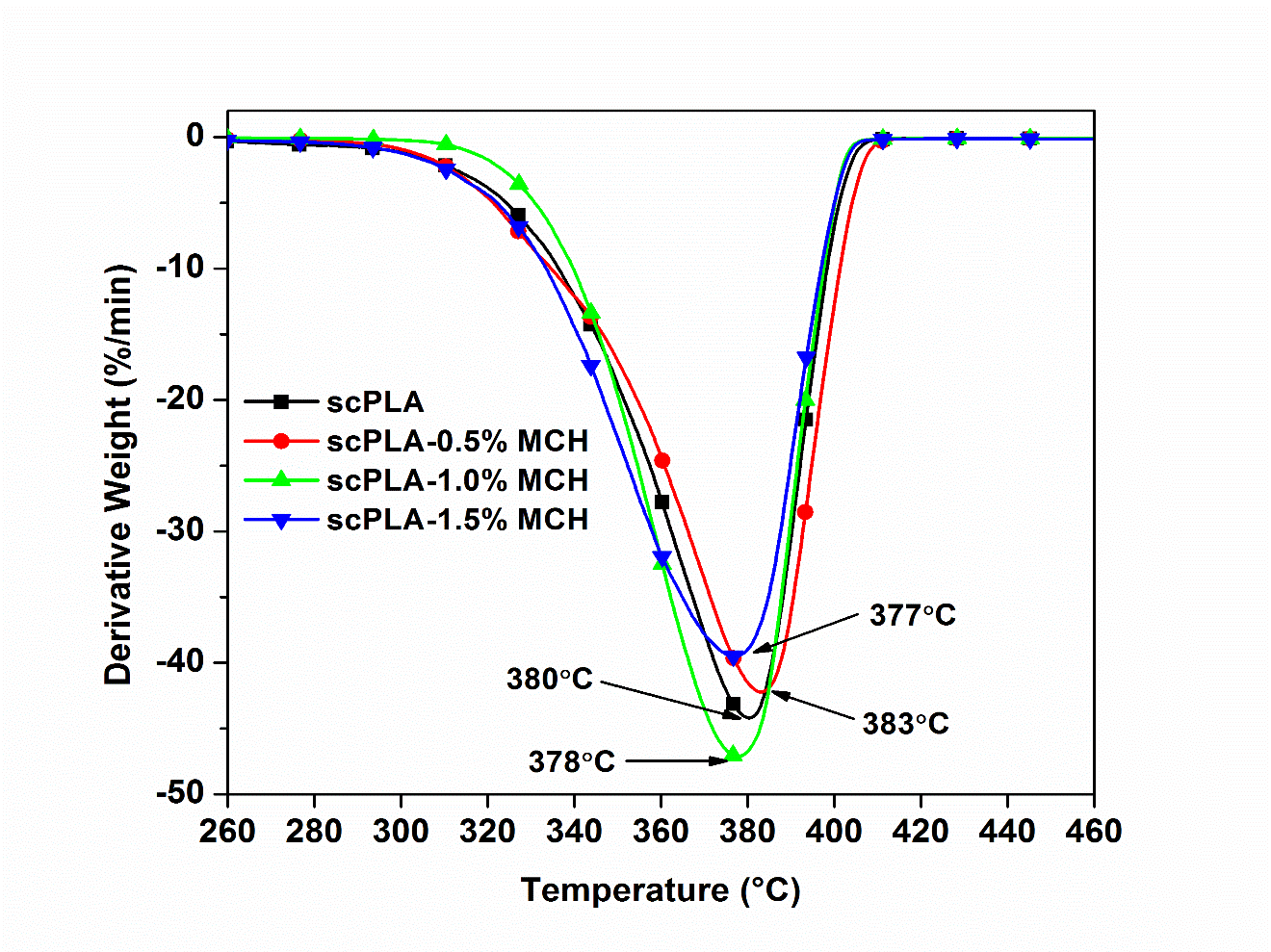

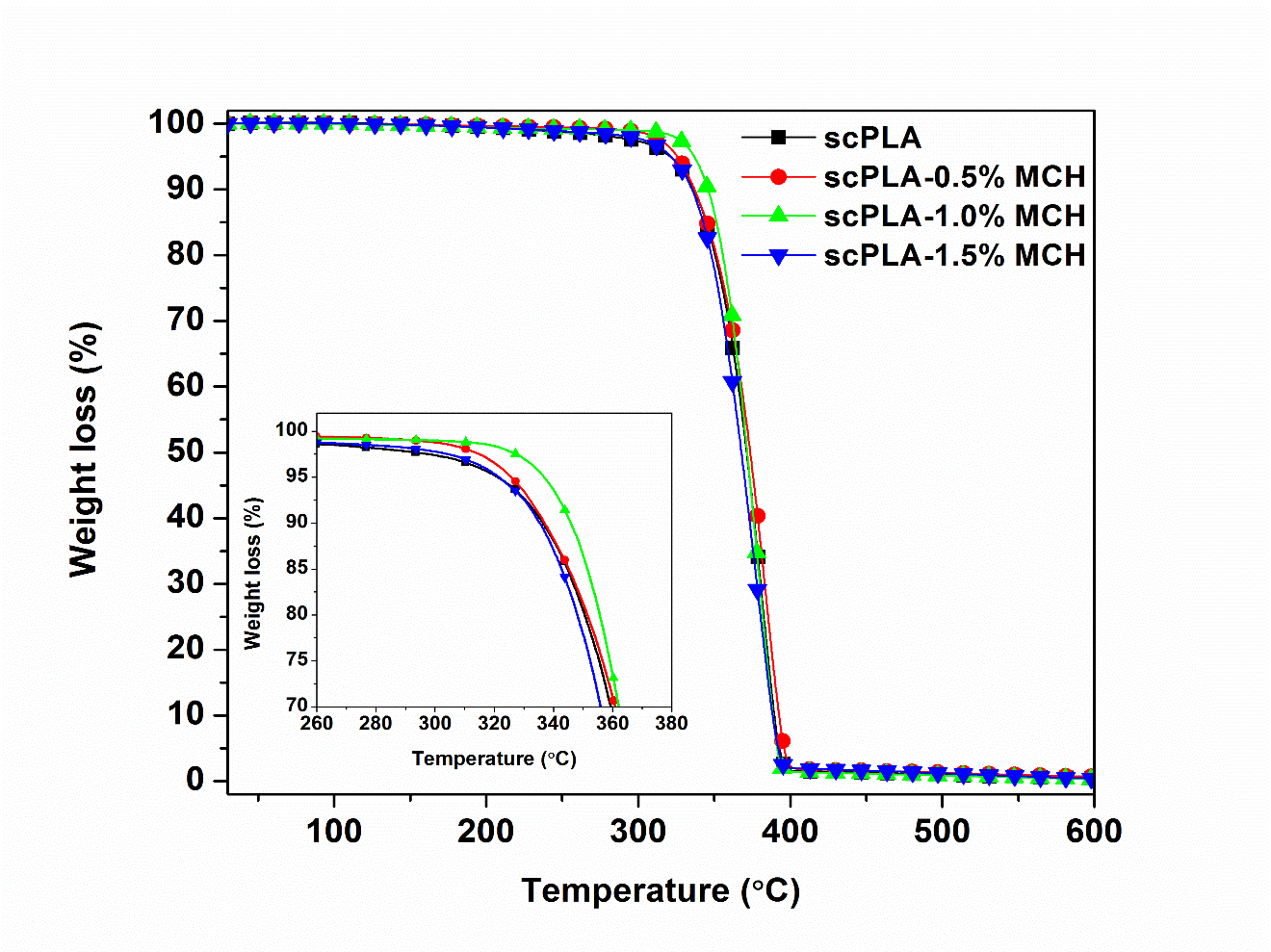


**(b)**

**(a)**

**Figure S8**: Thermal gravimetric analysis of scPLA with varying percentage of MCH (a), the derivative weight corresponds to the temperature (b)

**Table S1: Thermal properties of scPLA and biocomposites with various amount of MCH**

| **Sample Name** | **Heating rate (°C.min^-1^)** | | **T_g_ (°C)** | **T_m,hc_**  **(°C)** | **ΔH_m,hc_**  **(J.g^-1^)** | **T_m,sc_ (°C)** | **ΔH_m,sc_ (J.g^-1^)** |
| --- | --- | --- | --- | --- | --- | --- | --- |
| scPLA | 02 | 53.8 | | -- | -- | 192.9 | 20.56 |
| scPLA-0.5%MCH |  | 55.9 | | -- | -- | 207.2 | 40.93 |
| scPLA-1.0%MCH |  | 55 | | -- | -- | 207.6 | 39.35 |
| scPLA-1.5%MCH |  | 56.9 | | -- | -- | 206.2 | 30.2 |
| scPLA | 05 | 53.5 | | -- | -- | 199.8 | 34.17 |
| scPLA-0.5%MCH |  | 57.7 | | 148.2, 166.1 | 3.67, 2.4 | 209.0 | 46.71 |
| scPLA-1.0%MCH |  | 57.2 | | 166 | 2.32 | 208.8 | 42.69 |
| scPLA-1.5%MCH |  | 57.6 | | 150.3, 166.5 | 4.55, 2.87 | 208.5 | 28.12 |
| scPLA | 10 | 54.5 | | -- | -- | 201.2 | 34.02 |
| scPLA-0.5%MCH |  | 58.5 | | 149.3, 167.7 | 9.6, 8.4 | 208.6 | 39.9 |
| scPLA-1.0%MCH |  | 58.6 | | 167.2 | 5.42 | 210.2 | 36.31 |
| scPLA-1.5%MCH |  | 58.6 | | 169.3 | 5.11 | 209.2 | 22.28 |
| scPLA | 15 | 54.1 | | -- | -- | 201.8 | 40.8 |
| scPLA-0.5%MCH |  | 58.3 | | 165.4 | 3.85 | 209.8 | 39.72 |
| scPLA-1.0%MCH |  | 58.4 | | 165.9 | 7.29 | 209.3 | 37.91 |
| scPLA-1.5%MCH |  | 58.7 | | 165.5 | 5.57 | 207.4 | 30.55 |

**Kinetics and thermodynamics parameter calculation**

The growth of the spherulites is dependent on the crystallization temperature. The relation between temperature and growth rate is given by Lauritzen and Hoffmann[^1^](#_ENREF_1) which is expressed as:


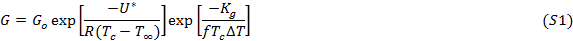


where, T_∞_ is the hypothetical temperature below which viscous flow ceases (T_∞_ = T_g_– 30 K), U^*^ is the activation energy for the segmental diffusion to the crystallization site from melt (U^*^ = 6300 J.mol^-1^),
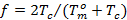
 is the correction factor, K_g_ is the nucleation constant, G is the growth rate of spherulite, G_o_ is a pre‑exponential factor, T_c_ is the crystallization temperature, ΔT is the degree of super‑cooling (
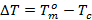
).

It is known that the crystallization rate is controlled by the nucleation which relates with the free energy for the formation of stable crystallites. The plot of Lauritzen and Hoffmann equation give the value of K_g_ which is related to the nucleation activity of fillers. The K_g_ value can be related and used to calculate the free energy of surface of lamella. The constants required for the calculation are taken from the literature available[^2-4^](#_ENREF_2) as U* = 6300 J.mole^-1^, a_o_ = b_o_ = 14.98Å, Equilibrium melting temperature = 243°C = 516.16 K, Glass transition temperature = 61°C = 334.16 K, ρ_c_ = 1.27 g.cm^-3^, Equilibrium melting enthalpy = 142 J.g^-1^, Heat of fusion per unit volume: 180.34 J.cm^-3^, k = 1.381×10^‑23^ J.K^-1^.

The nucleation constant (K_g_) is related to the surface free energy and is expressed as[^5^](#_ENREF_5)


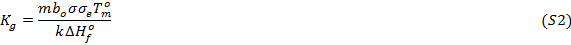


where, b_o_ is the thickness of monomolecular layer in the crystal, σ and σ_e_ are the lateral and folding surface free energies, respectively,
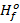
 is the heat of fusion per unit volume (
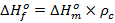
),
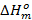
 is equilibrium melting enthalpy,
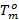
 is equilibrium melting temperature, ρ_c_ is the crystal density of polymer (stereocomplex PLA = 1.27 g.cm^-3^)[^2^](#_ENREF_2), k is the Boltzmann constant, m is the constant dependent on the regime of crystallization.

As the size of spherulites is too small and measuring the growth rate is quite difficult, the half time (t_1/2_) for the crystallization can replace the spherulite growth rate (G)[^5^](#_ENREF_5). It is believed that the nucleation rate is found to be maximum at t_1/2_. Therefore, the equation (1) can be written as:


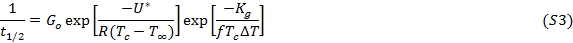


Equation (3) can be arranged by taking natural logarithm as


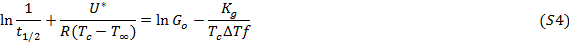


The plot between
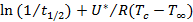
 versus
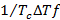
 will give strength line and slope of the line is calculated as value of K_g_ for the biocomposites.

The energy required for the formation of critical size of nucleus or the free energy of nucleation (ΔG^*^)[^6^](#_ENREF_6) can be calculated from the given equation:


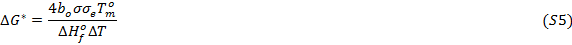


Samples are taken into the platinum crucible with lid and kept in the chamber with nitrogen flow of 20 mL.min^-1^. The sample is heated to 240°C with the rate of 50°C.min^-1^ and kept at same temperature for 5 min and then cooled to desired crystallization temperature with the rate of 50°C.min^-1^. The sample is kept at crystallization temperature isothermally for sufficient time for complete crystallization.


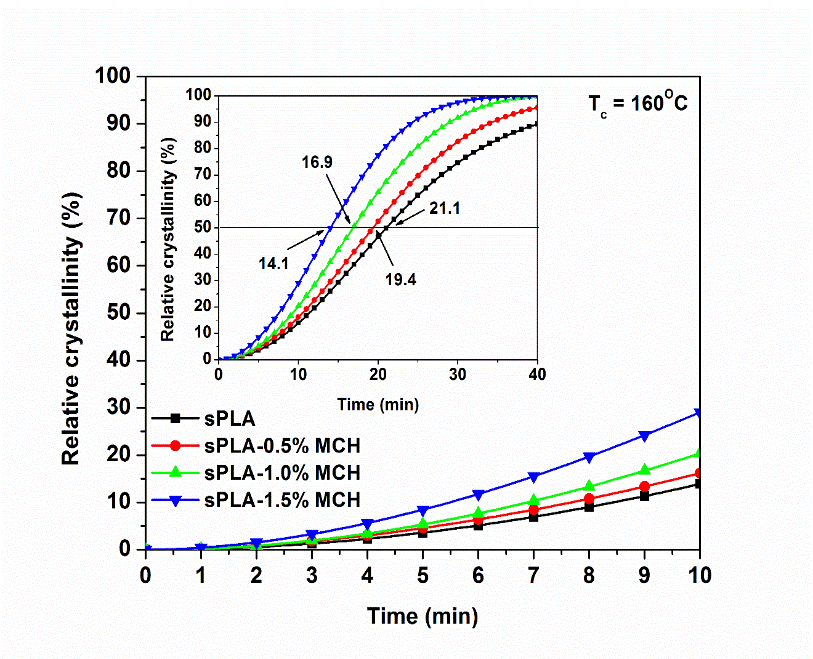

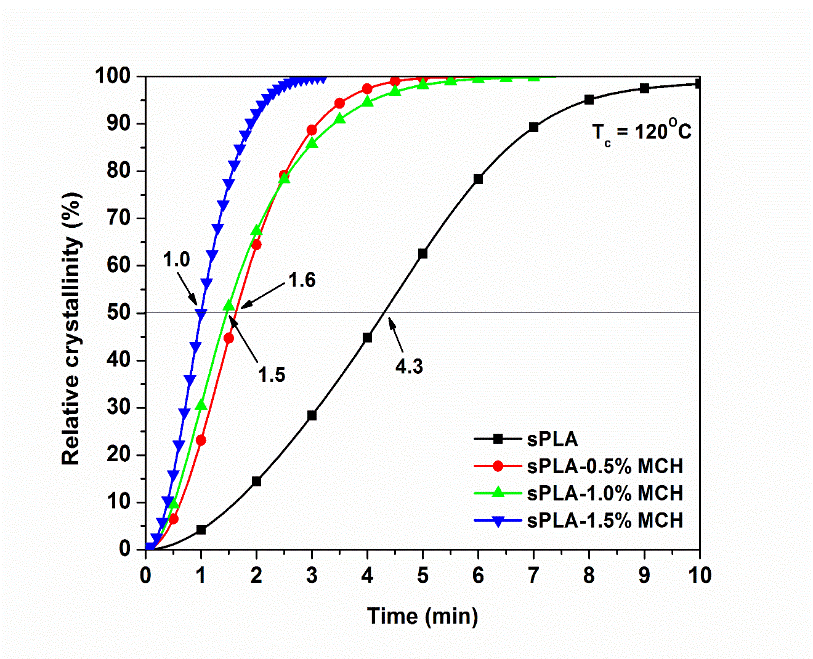

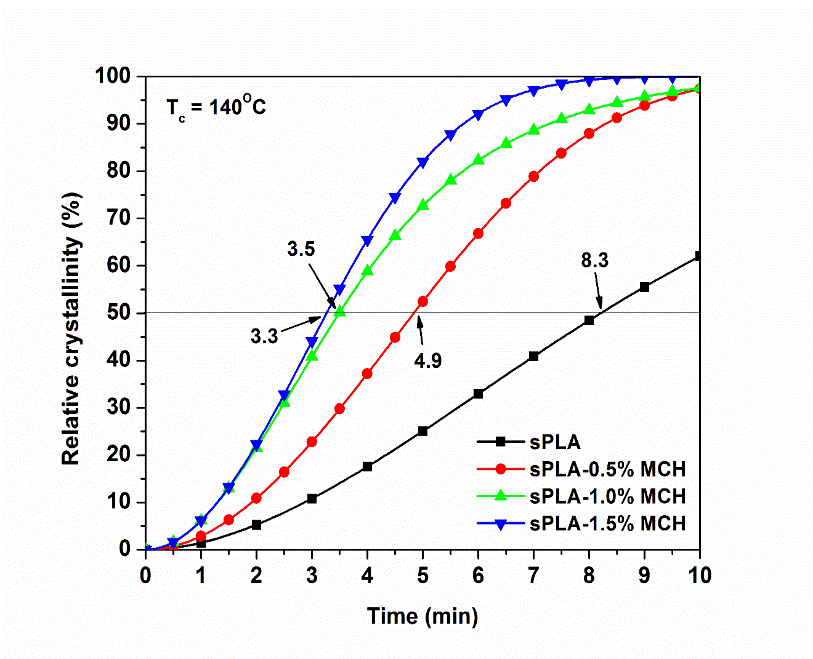


**Figure S9:** Relative crystallinity and t_1/2_ of sPLA and sPLA-MCH biocomposite at 120°C, 140°C and 160°C.

**Table S2:** Thermodynamic and kinetic parameters for the isothermal crystallization of sPLA‑MCH biocomposite.

| **Sample Name** | **T_c_ (°C)** | **t_1/2_ (min)** | **K_g_ (K^2^)** | **σσ_e_ (erg^2^.cm^-4^)** | **ΔG* (erg)** |
| --- | --- | --- | --- | --- | --- |
| **sPLA** | 120 | 4.3 | 6.3×10^5^ | 507.7 | 7.1×10^-13^ |
|  | 140 | 8.3 |  |  |  |
|  | 160 | 21.1 |  |  |  |
| **sPLA-0.5%MCH** | 120 | 1.6 | 7.6×10^5^ | 614.3 | 8.6×10^-13^ |
|  | 140 | 4.9 |  |  |  |
|  | 160 | 19.4 |  |  |  |
| **sPLA-1.0%MCH** | 120 | 1.5 | 7.6×10^5^ | 614.0 | 8.6×10^-13^ |
|  | 140 | 3.5 |  |  |  |
|  | 160 | 16.9 |  |  |  |
| **sPLA-1.5%MCH** | 120 | 1.0 | 7.7×10^5^ | 624.0 | 8.7×10^-13^ |
|  | 140 | 3.3 |  |  |  |
|  | 160 | 14.1 |  |  |  |
| T_c_: Crystallization temperature, K_g_ :Nucleation constant, σσ_e_ is the product of lateral and folding surface free energies, ΔG*: Free energy of nucleation, t_1/2_: Half time for the crystallization | | | | | |

**References**

1 Hoffman, J. D., Davis, G. T. & Lauritzen, J. I. in *Treatise on Solid State Chemistry: Volume 3 Crystalline and Noncrystalline Solids* (ed N. B. Hannay) 497-614 (Springer US, 1976).

2 Cartier, L., Okihara, T. & Lotz, B. Triangular Polymer Single Crystals:  Stereocomplexes, Twins, and Frustrated Structures. *Macromolecules* **30**, 6313-6322, doi:10.1021/ma9707998 (1997).

3 Garlotta, D. A Literature Review of Poly(Lactic Acid). *Journal of Polymers and the Environment* **9**, 63-84, doi:10.1023/a:1020200822435 (2001).

4 Jiang, L. *et al.* Stereocomplexation kinetics of enantiomeric poly(l-lactide)/poly(d-lactide) blends seeded by nanocrystalline cellulose. *RSC Advances* **5**, 71115-71119, doi:10.1039/c5ra12586c (2015).

5 Zhang, Q., Zhang, Z., Zhang, H. & Mo, Z. Isothermal and nonisothermal crystallization kinetics of nylon-46. *Journal of Polymer Science Part B: Polymer Physics* **40**, 1784-1793, doi:10.1002/polb.10237 (2002).

6 Naffakh, M., Marco, C. & Gómez-Fatou, M. A. Isothermal Crystallization Kinetics of Novel Isotactic Polypropylene/MoS2 Inorganic Nanotube Nanocomposites. *The Journal of Physical Chemistry B* **115**, 2248-2255, doi:10.1021/jp111965b (2011).
